# 3D chromatin remodeling potentiates transcriptional programs driving cell invasion

**DOI:** 10.1073/pnas.2203452119

**Published:** 2022-08-29

**Authors:** Benjamin Lebeau, Maïka Jangal, Tiejun Zhao, Cheng Kit Wong, Nolan Wong, Eduardo Cepeda Cañedo, Steven Hébert, Adriana Aguilar-Mahecha, Catherine Chabot, Marguerite Buchanan, Rachel Catterall, Luke McCaffrey, Geneviève Deblois, Claudia Kleinman, Morag Park, Mark Basik, Michael Witcher

**Affiliations:** ^a^Department of Experimental Medicine, Faculty of Medicine, McGill University, Montréal, QC H4A 3J1, Canada;; ^b^Lady Davis Research Institute, Jewish General Hospital, Montréal, QC H3T 1E2, Canada;; ^c^Department of Biochemistry, The University of British Columbia, Vancouver, BC V6T 1Z4, Canada;; ^d^Rosalind & Morris Goodman Cancer Research Centre, McGill University, Montréal, QC H3A 1A3, Canada;; ^e^Faculty of Pharmacy, Université de Montréal, Montréal, QC H3T 1J4, Canada;; ^f^Institute for Research in Immunology and Cancer, Université de Montréal, Montréal, QC H3T 1J4, Canada;; ^g^Department of Human Genetics, Faculty of Medicine, McGill University, Montréal, QC H4A 3J1, Canada;; ^h^Department of Biochemistry, McGill University, Montréal, QC H4A 3J1, Canada;; ^i^Department of Surgery, Faculty of Medicine, McGill University, Montréal, QC H4A 3J1, Canada;; ^j^Department of Oncology, Faculty of Medicine, McGill University, Montréal, QC H4A 3J1, Canada

**Keywords:** CTCF, subTAD, TAD, breast cancer, epigenetics

## Abstract

The contribution of deregulated chromatin architecture, including topologically associated domains (TADs), to cancer progression remains ambiguous. CCCTC-binding factor (CTCF) is a central regulator of higher-order chromatin structure that undergoes copy number loss in over half of all breast cancers, but the impact of this defect on epigenetic programming and chromatin architecture remains unclear. We find that under physiological conditions, CTCF organizes subTADs to limit the expression of oncogenic pathways, including phosphatidylinositol 3-kinase (PI3K) and cell adhesion networks. Loss of a single CTCF allele potentiates cell invasion through compromised chromatin insulation and a reorganization of chromatin architecture and histone programming that facilitates de novo promoter-enhancer contacts. However, this change in the higher-order chromatin landscape leads to a vulnerability to inhibitors of mTOR. These data support a model whereby subTAD reorganization drives both modification of histones at de novo enhancer-promoter contacts and transcriptional up-regulation of oncogenic transcriptional networks.

## Significance

During cancer progression, cells must adapt to facilitate invasion through stroma and subsequent metastatic growth in a distal niche. This adaption necessitates a great deal of plasticity, and there is evidence that epigenetic mechanisms govern these processes. While long-range chromatin interactions are key for proper genome organization and control of transcription, it is unclear whether reprogramming of three-dimensional chromatin architecture is important for mediating tumor progression. Here, we reveal that a common chromosomal aberration across cancers, CCCTC-binding factor (CTCF) copy number loss, potentiates cell invasion by reorganizing chromatin contacts at the level of sub–topologically associated domain (subTAD) interactions. We observe that subTAD reprogramming drives changes in gene expression that promote specific oncogenic pathways and predicts sensitivity to targeted therapy.

Hierarchical nuclear organization of chromatin plays essential roles during development and cell specification (1). As such, mapping and understanding the functionality of three-dimensional (3D) chromatin structure is now at the forefront of epigenetics research. Based on the advent of Hi-C sequencing technology (2), we know that the entire genome is partitioned into an assembly of topologically associated domains (TADs). TADs comprise 100-kb to 1-Mb regions of chromatin defined as a contiguous region enriched for DNA-DNA contacts between loci within the TAD, with few interactions outside of the TAD (3). TADs are commonly anchored by CCCTC-binding factor (CTCF), together with the cohesin complex, to establish a stable chromatin domain (4–6). Within TADs, smaller regions of self-interaction, called subTADs, add an additional layer of complexity to 3D chromatin architecture (7).

TADs and subTADs regulate gene transcription in mechanistically similar ways. By confining chromatin interactions to a particular region, they promote local interactions between *cis*-regulatory elements, such as enhancer-promoter interactions, while insulating the region from outside *cis*-regulatory elements. This allows for the specific pairings of promoters and enhancers required for proper temporal regulation of gene expression (8). Organization of chromatin into subTADs facilitates a more precise and dynamic local regulation of transcription than TADs alone would allow. Indeed, dynamic changes in subTAD organization drive transcriptional events of differentiation and cell identity, while TAD boundaries are mostly stable during these processes (1).

Proper TAD/subTAD organization is essential for temporal control of gene expression during development (9, 10). While aberrant activation of developmental programs appears to play an important role in tumor progression, it is unclear that widespread reorganization of chromatin domains is involved in this process. Despite evidence that altered TAD or subTAD organization locally at specific oncogenic loci may promote tumor initiation via aberrant changes to gene transcription (11), genomewide analysis of chromatin contacts using relevant models of tumor initiation and progression are clearly needed to provide further insights into a potential role of TAD reorganization in these processes.

Considering the central role of CTCF in maintenance of genomic TADs (12), it is not surprising that CTCF knockout (KO) leads to lethality at very early stages of embryonic development (13). Although not lethal, the loss of heterozygosity at the CTCF locus is also detrimental to cellular homeostasis (14), and CTCF appears to act as an haploinsufficent tumor suppressor gene (15, 16), with its loss impacting hematopoietic tumor initiation in CTCF hemizygous mice (15). In humans, Down syndrome–related acute megakaryoblastic leukemia carries CTCF deletions or mutations in 20% of all cases (17). Despite such clear evidence for a tumor suppressive role for CTCF in hematopoietic tissue, the importance of physiological levels and functionality of CTCF for the prevention of solid tumors remains ambiguous. Consistent with a putative tumor suppressor role, data from the Cancer Genome Atlas (TCGA) reveals that 63% of all breast tumors harbor CTCF copy number loss (CNL) (18). While it has been hypothesized that fluctuations in CTCF levels may impact chromatin looping, this has not been formally examined (19). Thus, it remains unclear whether transcriptional networks and topological features may be deregulated in breast epithelium undergoing CTCF CNL.

In the current study, we find that CTCF CNL in mammalian mammary epithelial cells potentiates subTAD reorganization and cell invasion. Likewise, reintroduction of CTCF into patient-derived xenograft (PDX) lines with low CTCF levels prohibits their invasion. We observe that restructuring of chromatin architecture, especially at the subTAD level, drives activation of the phosphatidylinositol 3-kinase (PI3K) pathway and overexpression of the classical oncogene SNAI1. These changes are associated with epigenetic reprogramming of Histone 3 lysine 27 acetylation (H3K27ac) and Histone 3 lysine 4 tri-methylation (H3K4me3) at regulatory regions. We also pose that these altered transcriptional events predict sensitivity to mammalian target of rapamycin (mTor) inhibitors (20) that potently repress the invasive capacity of cells carrying a single functional CTCF allele.

## Results

### Low CTCF Expression Promotes Invasiveness in Diverse Breast Cancer Models.

**Fig. 1.**
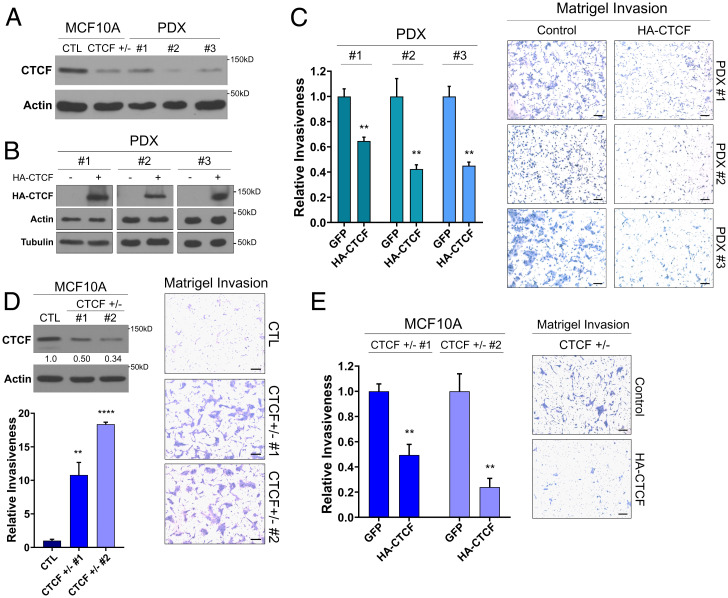
CTCF loss of heterozygosity promotes invasiveness and unorganized growth in distinct breast epithelial models. (*A*) Western blot showing low levels of CTCF, similar to the CTCF^+/−^ MCF10A, in the PDX cells. Loading CTL: actin. (*B*) Western blot of ectopic HA-CTCF expression in PDX cell lines. Loading CTLs: actin and tubulin. (*C*) Decrease in relative invasiveness of HA-CTCF PDXs to their respective GFP CTLs (mean ± standard error of mean (SEM]). *P* = 0.0031, 0.0084, and 0.0015 for PDX 1, 2, and 3, respectively. (*D*) Western blot of low CTCF levels in CTCF^+/−^ compared to CTL MCF10A. Quantification of relative CTCF band intensity in CTCF^+/−^ to CTL. Loading CTL: actin. Bar chart of the increased relative invasiveness of CTCF^+/−^ to CTL (mean ± SEM). *P* = 0.0066 and *P* < 0.0001 for CTCF^+/−^ 1 and 2, respectively. (*E*) Decrease in relative invasiveness of CTCF^+/−^ MCF10A with HA-CTCF addback to their respective GFP CTLs (mean ± SEM). *P* = 0.0013 for both CTCF^+/−^ 1 and 2. P value indicators, *P = 0.05, **P = 0.01, ***P = 0.001, ****P = 0.0001. See also *SI Appendix*, Fig. S1.

CTCF single allele deletions are prevalent in a majority of breast tumors (18). To better understand the consequences of this genetic aberration, we first surveyed conditionally reprogrammed cell lines from PDXs from triple-negative breast cancer patients harboring loss of one allele of CTCF (*SI Appendix*, Fig. S1*A*) and with CTCF expression equivalent to, or lower than in, our previously established CTCF^+/−^ MCF10A mammary epithelial cell line harboring a KO of one CTCF allele (21) (Fig. 1*A*). The low levels of CTCF in our cell lines tightly mimic those observed in vivo from a panel of tumor xenografts derived from a distinct set of triple-negative breast cancer patient tumors (*SI Appendix*, Fig. S1*B*). Thus, these data support our in vitro models as relevant systems to study the effects of low CTCF on oncogenic phenotypes.

Multiple clinical reports link 16q22.1 deletion, where CTCF resides, with metastasis (22, 23). Therefore, we investigated the impact of altered CTCF levels on the invasive capacity of cells, a critical step in cancer progression. For this, we employed Matrigel transwell invasion assays and CTCF addback to our PDX cell lines carrying low levels of CTCF. Following lentiviral addback of hemagglutinin-tagged CTCF (HA-CTCF, Fig. 1*B*), the increased CTCF expression led to reduced invasiveness of all three PDX cell lines tested (*P* = 0.0031, 0.0084, and 0.0015 for HA-CTCF 1 to HA-CTCF 3 against their respective control [CTL]) (Fig. 1*C*), despite the varied mutational background of each cell line. Next, we validated the impact of CTCF levels on invasiveness by comparing the effects of short hairpin RNAs (shRNAs) against CTCF (shCTCFs) or scrambled shRNA (shCTL) in MCF7 cells, a widely used, CTCF wild-type (WT) breast cancer cell line. Consistent with the inhibition of invasiveness brought about by CTCF addbacks, the reduction in CTCF levels resultant from the shCTCFs (*SI Appendix*, Fig. S1*C*) led to a significant increase in MCF7 invasiveness compared to shCTL (*P* = 0.021 and 0.0051 for shCTCF 1 and 2) (*SI Appendix*, Fig. S1*C*). Altogether, these studies confirm a relationship between low CTCF expression and increased invasiveness in distinct breast cancer models.

### CTCF Single Allele KO Induces Oncogenic Phenotypes in Mammary Epithelial Cells.

Next, to study the effect of altered CTCF expression in mammary tissue independently of the heavy mutational burden of breast cancer models, we investigated the effect of low CTCF expression in the nontransformed breast epithelial cell line MCF10A carrying a single allele KO of CTCF (MCF10A-CTCF^+/−^) via CRISPR-Cas9 (21) (Fig. 1*D*).

Using this model, we screened for several classical oncogenic phenotypes, including cell invasion, altered morphology, increased proliferation, and deregulated mammosphere growth. Similar to what we observed in breast cancer models, the loss of one copy of CTCF increased the capacity of MCF10A to invade through a Matrigel matrix. CTCF^+/−^ cells readily invaded through Matrigel at a rate significantly higher than CTCF^+/+^ CTL MCF10A cells (*P* = 0.0066 and *P* < 0.0001 for CTCF^+/−^ 1 and 2) (Fig. 1*D*). To support a direct link between the loss of CTCF and the acquired invasiveness, we carried out lentiviral-mediated addback of HA-CTCF within our MCF10A models. As in the PDX cell lines, the restoration of CTCF levels was able to significantly reduce the invasiveness of CTCF^+/−^ cells (Fig. 1*E* and *SI Appendix*, Fig. S1*D*). Phenotypically, the morphology of the CTCF^+/−^ cells in two-dimensional (2D) culture was markedly similar (*SI Appendix*, Fig. S1*E*), while their proliferation rate was slightly reduced compared to CTL cells (*SI Appendix*, Fig. S1*F*). MCF10As spontaneously form organized hollow ductal acinar-like structures in 3D culture (24), so we investigated the impact of lowered CTCF levels on growth in this system. Strikingly, CTCF^+/−^ MCF10A acini form significantly larger, less hollow (*P* < 0.0001), and structurally deformed (*SI Appendix*, Fig. S1 *G* and *F*) mammospheres compared to CTL counterparts. Together, these results point toward a potentially important role for the loss of heterozygosity of CTCF in cancer progression, as it promotes disorganized 3D growth and invasiveness, two strongly linked oncogenic abilities critical for tumors to progress from benign to advanced stages of cancer.

### CRISPR-Cas9 KO of CDH1 Loss Does Not Phenocopy CTCF Single Allele KO.

The loss of 16q22.1 in cancer commonly encompasses multiple genes, notably the tumor suppressor gene CDH1, whose mutation may play a role in lobular carcinoma of the breast (25). To compare CDH1 loss with that of CTCF, we generated CDH1 KO MCF10A cells using the same CRISPR-Cas9 protocol as the one we used to establish the MCF10A-CTCF^+/−^ line (*SI Appendix*, Fig. S1*I*). In stark contrast to CTCF^+/−^ cells, the invasiveness of the CDH1 KO was not increased (*SI Appendix*, Fig. S1*I*). These data validate the relevance and specificity of our CTCF^+/−^ model, independent of CRISPR-Cas9 artifacts.

### Reprogramming of Transcriptional Networks Leads to Activation of Oncogenic Signaling in CTCF^+/−^ Cells.

**Fig. 2.**
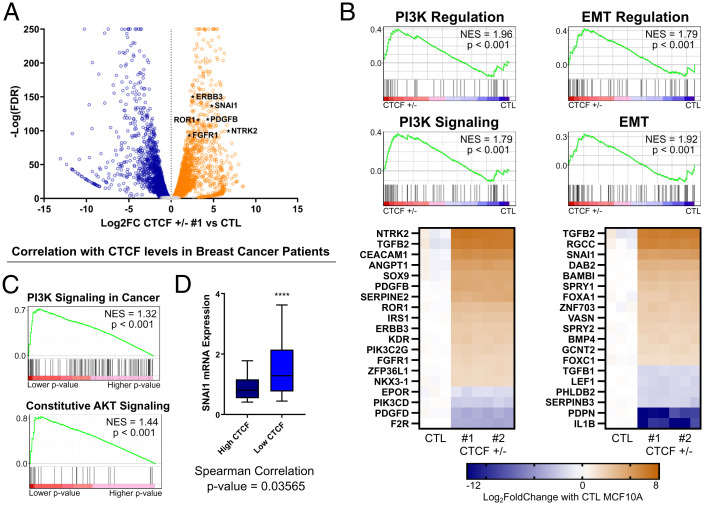
RNA-seq reveals oncogenic expression underlying the invasive phenotypes. (*A*) Volcano plot of transcriptomic changes between CTCF^+/−^ 1 and CTL MCF10A. Genes of the PI3K and EMT pathways, marked as black stars, are among the top up-regulated genes. (*B*) *Top:* GSEA of Gene Ontology PI3K pathways and EMT pathways up-regulated in CTCF^+/−^ MCF10A compared to CTL. *Bottom:* Heatmaps of the top 20 most up- or down-regulated genes, ranked by abs(log2FC), in the PI3K and EMT regulation pathways. (*C*) Most enriched pathway by *P* value (PI3K signaling) and NES (Akt signaling) following GSEA prerank analysis of genes significantly correlating with CTCF expression in TCGA breast cancer patient RNA-seq data. Genes were ranked by −log(Spearman test *P* value). (*D*) Box plot (10th to 90th percentile) of higher SNAI1 expression levels in low-CTCF breast tumors (*P* < 0.0001, one-tailed Student’s *t* test) detected by RNA-seq in TCGA breast cancer patients for tumors in the top 20% of low CTCF expression compared to the top 20% of high CTCF expression. The *P* value for the Spearman correlation test is also noted. Scale bars represent distribution or mRNAs, from minimum (low bar) to maiximum (upper bar). See also *SI Appendix*, Fig. S2.

To gain insight into the mechanism whereby CTCF^+/−^ cells acquire the oncogenic phenotypes described above, we carried out ribonucleic acid sequencing (RNA-seq) to compare global gene expression profiles of CTL and CTCF^+/−^ MCF10A cells. Using DESeq2 (26), we detected 2,976 and 2,893 genes that were significantly transcriptionally altered in CTCF^+/−^ 1 and 2, respectively, compared to CTL [basemean > 100, absolute log2 fold-change (abs(log2FC)) >1, adjusted *P* < 0.05]. The transcriptional changes were highly reproducible, as the respective changes in gene expression in each CTCF^+/−^ clone compared to CTL correlated very strongly (*r* = 0.9937, *P* < 0.0001) (*SI Appendix*, Fig. S2*A*). Of the 2,765 genes commonly altered in both CTCF^+/−^ clones, a slight majority of 1,503 genes were up-regulated (54%), compared 1,261 genes that were down-regulated (46%) (Fig. 2*A*). These results demonstrate that a specific subset of genes is consistently sensitive to CTCF depletion in breast epithelial cells.

GSEA analysis of the RNA-seq data revealed that multiple gene sets related to the phosphoinositide 3-kinase (PI3K) and epithelial-to-mesenchymal transition (EMT) pathways were strongly up-regulated in the CTCF^+/−^ cells (Fig. 2*B*). Indeed, Gene Ontology “positive regulation of phosphatidylinositol-3-kinase signaling” and “epithelial-to-mesenchymal transition” were in the top 5% and 7% by normalized enrichment score (NES) in our gene set enrichment analysis (GSEA), respectively, out of 839 significantly up-regulated pathways. Similarly, gene sets related to these pathways were consistently among the top 10 enriched pathways using kyto enrichment of genes and genomes (KEGG), Reactome, or protein analysis trough evolutionary relationships (PANTHER) pathway analysis tools and distinct ranking methods (*SI Appendix*, Fig. S2*B*). The PI3K pathway is a classical oncogenic pathway aberrantly activated in diverse cancers that drives both invasiveness and altered mammosphere morphology (24). Among the top up-regulated genes in our CTCF^+/−^ clones were ERBB3 and FGFR1, well-characterized receptor tyrosine kinases and oncogenes that activate the phosphorylation cascade of the PI3K pathway (27). PI3K signaling feeds into the EMT pathway, partially through translational up-regulation of classical oncogenes such as SNAI1, which itself promotes invasion (28). Interestingly, CTCF sites surrounding SNAI1 are enriched for noncoding mutations in cancer (29), and SNAI1 was among the top hits within the EMT pathway based on our RNA-seq data (Fig. 2*B*). Consistent with an up-regulation of EMT-related genes, such as SNAI1 (30), and our invasive phenotype, down-regulated genes were enriched for those involved in the promotion of cell-to-cell contact, such as cellular adhesion pathways (*SI Appendix*, Fig. S2*C*). The marked up-regulation and down-regulation of top hits were validated using qPCR (*SI Appendix*, Fig. S2*D*). Considering that CTCF^+/−^ cells do not undergo obvious changes in morphology, it is likely that these cells undergo a partial EMT that is reversible upon re-expression of CTCF (Fig. 1*E*).

Next, we investigated RNA-seq data from the TCGA database of breast cancer patients. To detect groups of genes whose expression may be altered by varying CTCF levels, we computed the statistical correlation between each gene and CTCF expression in each patient. We then carried out gene ranking based on Spearman test *P* values coupled with GSEA prerank pathway analysis. Similar to what we observed in MCF10A cells, pathways involved in PI3K signaling were overrepresented in the top 10 altered pathways by NES (*SI Appendix*, Fig. S2*E*) and *P* values (Fig. 2*C*). Interestingly, the association between high SNAI1 expression and low CTCF has also been observed clinically, as we found a significant correlation between low CTCF and high SNAI1 expression in patients’ breast tumors (Fig. 2*D*). Overall, these results imply a role for CTCF in the regulation of genes involved in the PI3K signaling pathway gene and important regulator of EMT, such as SNAI1.

### Activation of the PI3K Pathway following CTCF CNL.

**Fig. 3.**
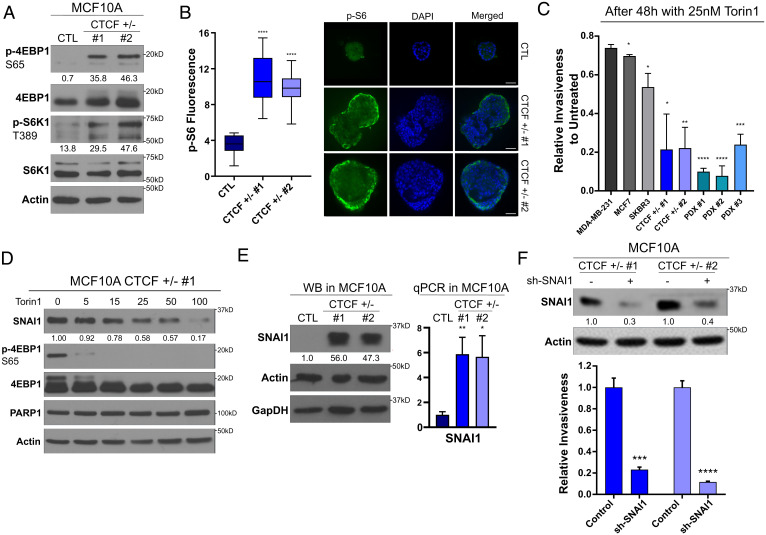
The PI3K pathway and SNAI1 are central for the oncogenic properties of CTCF^+/−^ cells. * values for *P* values as indicated in Fig. 1. (*A*) Western blot showing maintained phosphorylation of mTORC1 targets under serum-free conditions in CTCF^+/−^ cells. Quantification represents the band intensity normalized on background. Loading CTL: actin. (*B*) Mammosphere immunofluorescence and quantification of increased S6 fluorescence of the outer layer of the mammosphere in CTCF^+/−^ compared to CTL MCF10A. Bars represent minimum and maximum values. *P* < 0.0001 for CTCF^+/−^ 1 and 2 compared to CTL. (*C*) Invasiveness following 48 h of 25 nM Torin1 treatment normalized relative to the untreated invasiveness of each cell line (mean ± SEM). *P* values comparing each cell line to the relative invasiveness of MDA-MB-231: MCF7 = 0.031508, SKBR3 = 0.019340, CTCF^+/−^ 1 = 0.018925, CTCF^+/−^ 2 = 0.003089, PDX 1 = 0.000002, PDX 2 = 0.000076, and PDX 3 = 0.000262. (*D*) Western blot, using serum-free conditions, for SNAI1 levels and 4EBP1 phosphorylation following 24 h of Torin1 treatment. Quantification of SNAI1 band intensity relative to untreated levels is shown below each blot. Loading CTLs: PARP1 and actin. (*E*) Western blot (WB) of SNAI1 levels in CTCF^+/−^ MCF10A cells. Quantification of relative SNAI1 band intensity to CTL is shown below the topmost blot. Loading CTLs: actin and GapDH. Bar chart (mean ± SEM) of qPCR validation of SNAI1 overexpression at the mRNA levels. (*F*) Western blot of SNAI1 levels following anti-SNAI1 short hairpin RNA treatment. *Bottom:* Quantification of relative SNAI1 band intensity to shCTL. Loading CTL: actin. Bar chart of decreased relative invasiveness of the shSNAI1-treated CTCF^+/−^ MCF10A compared to shCTL treated (mean ± SEM). *P* = 0.00103 and *P* = 0.000141 for CTCF^+/−^ 1 and CTCF^+/−^ 2 respectively. All *P* values were calculated using Student’s *t* test.

Now guided by our RNA-seq data, we examined whether the PI3K pathway was hyperactivated in the CTCF^+/−^ MCF10A cells to determine whether this signaling contributes to the invasive phenotype. First, we screened for increased activation of key downstream effectors of PI3K signaling, including phosphorylation of 4EBP1 (serine 65) and S6K1 (threonine 389), direct targets of the mTORC1 complex (31). Under conditions of serum starvation, where phosphorylation of S6K1 and 4EBP1 were weakly detected in the CTL cells, a strong phosphorylation signal was detected in the CTCF^+/−^ cells (Fig. 3*A*). As the up-regulation of the PI3K pathway can alter the morphology of mammospheres (24), we tested whether the elevated activation of PI3K might be observed under 3D culture conditions. Similarly, we detected a pronounced phosphorylation of S6, the direct target of S6K1, in both CTCF^+/−^ mammosphere populations, while it was absent from the CTL acini (Fig. 3*B*). Since the outer region of mammospheres is expected to be the primary proliferative zone due to the accessibility of nutrients and oxygen, we developed a custom script to visually isolate and quantify the fluorescence of this region for individual mammospheres. We detected that phosphorylation of S6 was 3.1 and 2.7 times higher in CTCF^+/−^ 1 and 2 than in the CTL acini (*P* < 0.0001). Thus, under conditions of both 2D and 3D culture, reduced pools of CTCF lead to transcriptional reprogramming that activates the PI3K pathway.

Based on the aberrant activation of PI3K signaling in CTCF^+/−^ cells, we surmised that their invasivity may be vulnerable to inhibitors of this pathway. We targeted mTORC1/2 because these kinase complexes assimilate the signals from diverse branches of the PI3K signaling cascade (31). We carried out Matrigel transwell invasion assays, following a 48-h mTORC1/2 inhibition, using the second-generation mTor inhibitor Torin1 (32). Since CTL MCF10As are mostly noninvasive, we compared the changes in invasiveness of our CTCF^+/−^ MCF10A cells and PDX cell lines to the well-characterized breast cancer cell lines: MDA-MB-231, MCF7, and SKBR3. Following Torin1 treatment at 25 nM, both CTCF^+/−^ and CTCF-low MCF10A PDX lines were markedly sensitive to these low concentrations, being significantly more repressed in their ability to invade than the trio of breast cancer cell lines carrying higher CTCF levels (Fig. 3*C*). These data indicate that the PI3K pathway plays a central role in driving the invasion of normal epithelial cells with reduced CTCF levels, while late-stage triple-negative breast cancer (TNBC) lines such as MDA-MB-231 may utilize multiple, or alternative, pathways to achieve this phenotype.

We also validated that low concentrations of Torin1 treatment efficiently inhibited mTORC1. Concentrations as low as 5 nM strongly abrogate the phosphorylation of 4EBP1 in the CTCF^+/−^ cells under starved conditions (Fig. 3*D*). As the PI3K pathway has also been shown to control the protein expression of SNAI1 through translational up-regulation (28), we investigated the impact of Torin1 treatment on SNAI1 expression. We detected a marked, dose-dependent drop in SNAI1 protein levels following 24 h of Torin1 exposure (Fig. 3*D*). Since SNAI1 overexpression promotes invasiveness in multiple models (33) and it is strongly overexpressed at the messenger RNA (mRNA) and protein levels in our CTCF^+/−^ MCF10A (Fig. 3*E*), we decided to investigate whether it is an important downstream target of PI3K and playing a role in the invasiveness of the CTCF^+/−^ cells. To do so, we used lentiviral-mediated shRNA knockdown of SNAI1 and surveyed the changes to cell invasion. The down-regulation of SNAI1 led to a significant reduction of both SNAI1 protein levels and CTCF^+/−^ invasiveness (Fig. 3*F*). Overall, these results highlight the importance of the up-regulation of the PI3K pathway and its downstream effector, SNAI1, for the oncogenicity of the CTCF^+/−^ cells. These indicate that the invasion of tumors harboring CTCF CNL, coupled with elevated SNAI1, may be susceptible to therapeutic intervention with inhibitors of mTORC1/2.

### Low CTCF Expression Alters Its Binding to DNA Surrounding Oncogenes.

**Fig. 4.**
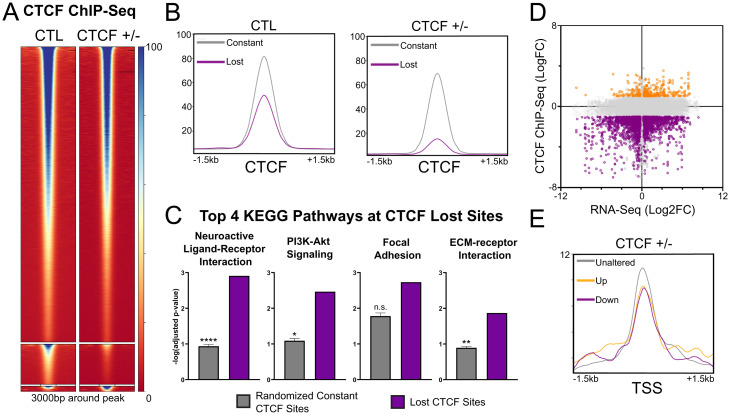
CTCF depletion alters the CTCF DNA binding pattern. (*A*) CTCF ChIP-seq heatmap of constant, lost, and gained sites (*Top* to *Bottom*). (*B*) Reduced average CTCF ChIP-seq read density of lost sites compared to constant sites in the CTL and CTCF^+/−^ MCF10A. (*C*) Differential enrichment of the top 4 KEGG pathways, dominated by PI3K- and ECM-related pathways (ranked by geneRatio), at lost sites of CTCF compared to 100 equinumerous subsets of constant sites (mean ± SEM). n.s. = not significant (*D*) Dot plot of gene expression (log2FC) and CTCF binding (logFC) changes between CTCF^+/−^ and CTL MCF10A for binding sites colocalizing (±3 kb) with expressed genes. Lost sites (purple) are found in proximity to both up- and down-regulated genes. Gained sites (orange) are differentially found in proximity to up-regulated genes. (*E*) Decreased average CTCF ChIP-seq read density in CTCF^+/−^ MCF10A at the TSSs of all up-regulated genes (adjusted *P* < 0.05, log2FC > 1) and down-regulated genes (in purple, adjusted *P* < 0.05, log2FC < −1) compared to unaltered genes [adjusted *P* > 0.05, abs(log2FC) < 0.5]. See also *SI Appendix*, Fig. S3.

It is reasonable to expect that the reduced nuclear pool of CTCF would compromise the number of occupied CTCF sites on the chromatin. To gain mechanistic insight into the altered oncogenic transcriptional networks of CTCF^+/−^ MCF10A cells, we carried out chromatin immunoprecipitation sequencing (ChIP-seq) to map CTCF binding across the genome (Fig. 4*A*). We identified that a majority of CTCF binding sites, 38,775 out of the 44,802 peaks called by MACS2 (34), were left unchanged between the CTL and CTCF^+/−^ MCF10A cells. Considering that CTCF levels were reduced by ∼50% to 60% in these cells, it is clear that the nuclear pool of CTCF is in excess of that required for genomic regulation, consistent with previous reports showing that a significant fraction of CTCF is unbound within interphase cell populations (35). This excess is likely a safeguard against genomic instability and protection of the transcriptome that might stem from fluctuating CTCF levels. However, as expected, a subset of 5,313 sites displayed reduced or lost CTCF binding in CTCF^+/−^ MCF10As compared to the CTL (false discovery rate [FDR] < 0.01, logFC < −1). Surprisingly, a small cluster of 714 sites displayed a gain of CTCF binding (FDR < 0.01, logFC > 1) (*SI Appendix*, Fig. S3*A*).

Next, we investigated the differences in binding strength and distribution between the sites lost and constant in CTCF^+/−^ cells. The average read density was lower for sites within the lost cluster compared to the constant cluster in CTL MCF10As (Fig. 4*B*). The genomic distributions of lost and gained sites were also unique compared to constant sites. Compared to 19% of constant sites, 29% of CTCF lost sites were found on promoters, such as the promoter of SNAI1 (*SI Appendix*, Fig. S3 *B* and *C*). In contrast, 37% of lost sites, including a ERBB3 downstream site (*SI Appendix*, Fig. S3*D*), and 41% of constant sites were located in distal intergenic regions compared to 53% of gained sites (*SI Appendix*, Fig. S3*B*).

Consistent with our RNA-seq, KEGG pathway analysis of the CTCF binding sites within the lost cluster showed strong enrichment surrounding genes involved in the PI3K-Akt signaling pathway (ranked second by relative gene count). Multiple pathways related to cell mobility, such as extra-cellular matrix (ECM)--receptor activation (ranked fourth), were also observed, which displayed significant enrichment when compared to an equinumerous set of constant CTCF sites (Fig. 4*C*). These results suggest that a subset of weakly binding CTCF sites, showing enrichment around promoters and genes involved in the PI3K pathway and cell invasion, such as SNAI1, are more sensitive to CTCF depletion in mammary epithelial cells.

### CTCF Lost Sites Are Frequently Proximal to Deregulated Genes.

CTCF may impact gene transcription through binding proximal or distal to transcription start sites (TSSs) through multiple mechanisms. To gain further insights into the mechanisms whereby altered CTCF binding might impact transcriptional events in MCF10A cells carrying a single functional CTCF allele, we mapped lost sites to determine their proximity to TSSs. About half of all CTCF lost sites (2,408 out of 5,313) were found with proximity (±3 kb) to significantly altered genes (basemean > 100, adjusted *P* < 0.05). Interestingly, a significant fraction of lost sites was found around both strongly up-regulated (log2FC > 1,530 sites) and strongly down-regulated (log2FC < −1,716 sites) genes (Fig. 4*C*). This highlights the complexity of gene regulation mediated by CTCF but underscores that loss of CTCF binding frequently impacts the transcription of proximal loci. The intensity of the loss of CTCF significantly, but weakly, correlated with both up-regulation (r = −0.1, *P* = 0.0056) and down-regulation (*r* = 0.2, *P* < 0.0001) of gene expression. Similarly, both up-regulated and down-regulated genes displayed a slightly lower average CTCF ChIP-seq read density on their TSSs (Fig. 4*D*).

Regarding gained CTCF sites, of 714 such sites, 334 were proximal to 267 unique altered genes (adjusted *P* < 0.05). Of these genes, 192 (72%) were significantly up-regulated, but only 107 genes reached a log2FC ≥ 1. Pathway analysis of the 107 up-regulated genes or 267 significantly altered genes revealed no clear enrichment, as no pathways were significant by FDR. These results are expected when the number of genes is low and the distribution is primarily stochastic.

While proximal loss of CTCF is associated with both up-regulation and down-regulation of gene expression, it only mildly correlated with the intensity of altered gene expression. This hints that in many cases, the changes in transcription observed in CTCF heterozygous cells are likely driven through indirect, or downstream, mechanisms, including changes in chromatin conformation or epigenetic reprogramming that are potentiated by CTCF loss, but are likely not due to a loss of CTCF interaction with the core transcription machinery (36, 37).

### CTCF Loss Potentiates Epigenetic Reprogramming at Transcriptionally Altered Genes.

**Fig. 5.**
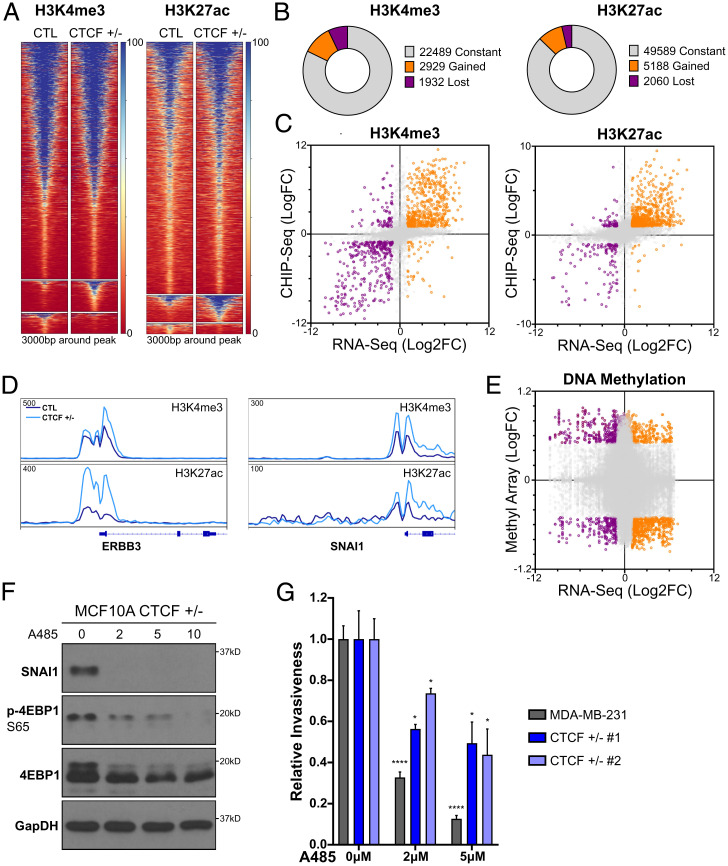
Epigenetic reprogramming of activating histone marks drives changes in gene expression. (*A*) H3K4me3 and H3K27ac ChIP-seq heatmaps for constant, gained, and lost sites (*Top* to *Bottom*). (*B*) Partitioning of constant, gained, and lost clusters from *A*. (*C*) Dot plot of highly correlating gene expression (log2FC) and H3K4me3 or H3K27ac (logFC) changes between CTCF^+/−^ and CTL MCF10A for binding sites colocalizing (±3 kb) with expressed genes. (*D*) ChIP-seq track of the normalized read density for H3K27ac or H3K4me3 surrounding ERBB3 and SNAI1. (*E*) Dot plot of gene expression (log2FC) and EPIC methyl array (logFC) changes between CTCF^+/−^ and CTL MCF10A for binding sites colocalizing (±3 kb) with expressed genes. (*F*) Western blot, under starved conditions, for 4EBP1 phosphorylation and SNAI1 levels following 48 h of HATi A485 treatment (in micromolars). (*G*) Relative invasiveness of A485-treated CTCF^+/−^ MCF10A and CTL MDA-MB-231 (mean ± SEM) . *P* values of treated compared to untreated cells; 2 μM A485: MDA-MB-231 < 0.0001, CTCF^+/−^ 1 = 0.0444, and CTCF^+/−^ 2 = 0.0232; 5 μM A485: MDA-MB-231 < 0.0001, CTCF^+/−^ 1 = 0.0284, and CTCF^+/− ^2 = 0.0252. See also *SI Appendix*, Fig. S4.

Destabilization of CTCF binding has been linked to numerous epigenetic changes (38, 39). Thus, we investigated whether the changes to gene expression and CTCF binding were associated with changes to chromatin marks. First, we screened for multiple activating and silencing histone marks on representative altered genes using ChIP-quantitative polymerase chain reaction (ChIP-qPCR). Although we detected significant changes in (H3K4me3) and H3K27ac associated with altered transcription (*SI Appendix*, Fig. S4 *A* and *B*), we did not detect strong changes with the repressive marks histone 3 lysine 27 trimethylation (H3K27me3) and histone 3 lysine 9 tri-methylation (H3K9me3) (*SI Appendix*, Fig. S4 *C* and *D*). This is consistent with a previous study where changes to CTCF binding across multiple genomes were not strongly linked to differences in H3K27me3 (38). Therefore, we proceeded to map H3K4me3 and H3K27ac genomewide using ChIP-seq to compare CTL MCF10A with CTCF^+/−^ cells (Fig. 5*A*). A majority of H3K4me3 (∼82%) and H3K27ac (∼87%) peaks were conserved between CTCF^+/−^ and CTL MCF10A. However, both H3K4me3 and H3K27ac showed significant alterations upon loss of CTCF. H3K4me3 and H3K27ac gained enrichment at 2,929 and 5,188 loci, respectively. Further, H3K4me3 was reduced at 1,932 sites and H3K27ac was reduced at 2,060 sites [abs(logFC) ≥ 1, FDR ≤ 0.05] (Fig. 5*B*). Overall, CTCF loss potentiated a gain of marks associated with gene activation. Then, we assessed whether these changes to histone marks correlated with altered gene expression. We observed a pronounced and statistically significant gain of H3K27ac at up-regulated genes (*r* = 0.64, *P* < 0.0001) (Fig. 5*C*), including oncogenes such as ERBB3 and SNAI1 (Fig. 5*D*), compared to a more modest correlation of H3K4me3 with up-regulated genes (*r* = 0.45, *P* < 0.0001) (Fig. 5*C* and *D*).

CTCF loss has been indirectly linked to deregulated DNA methylation (40, 41), and it is possible that altered DNA methylation contributes to transcriptomic changes observed in CTCF^+/−^ cells. We carried out bisulfite conversion and investigated the association between genomewide changes in DNA methylation and transcriptomic changes using the Illumina 850k methyl array with our RNA-seq data. Contrary to the strong correlation detected between changes in activating marks and gene expression, the changes in DNA methylation pattern observed in CTCF^+/−^ did not correlate with changes in gene expression (r = −0.04, *P* < 0.0001) (Fig. 5*E*). These results indicate that under conditions of subphysiological CTCF levels, changes in gene expression most specifically linked to global reprogramming of H3K27ac.

To test for a role of gained H3K27ac in the promotion of cell invasion, we treated CTCF^+/−^ cells with the histone acetyltransferase inhibitor (HATi) A485, that targets CREB-binding protein (CBP) (42). First, we validated the ability of A485 to inhibit the deposition of H3K27ac using western blotting (*SI Appendix*, Fig. S4*E*). Linking acetylation to the transcriptomic profiles defined on our CTCF^+/−^ MCF10A cells, under serum-starved conditions, A485 treatment efficiently resolves the hyperactivation of the PI3K/mTor pathway, as indicated by a dose-dependent reduction of 4EBP1 phosphorylation (Fig. 5*F*). Similarly, CBP inhibition blocked SNAI1 expression, linking the gain of H3K27ac to its up-regulation (Fig. 5*F*). As inhibition of both the PI3K pathway and SNAI1 expression reduced the invasiveness of the CTCF^+/−^ cells, we tested their invasivity after exposure to A485 treatment and further compared the effects with those observed in MDA-MB-231 cells. Similar to mTor inhibition and SNAI1 knockdown, A485 treatment significantly reduced the invasiveness of the CTCF^+/−^ cells (Fig. 5*G*), further supporting the hypothesis that the increased deposition of H3K27ac plays a key role in the oncogenic phenotypes caused by the loss of CTCF. Interestingly, MDA-MB-231 cells were noticeably sensitive to this treatment (Fig. 5*G*) as well. These results highlight a general dependency on increased histone acetylation during the invasion process of aggressive epithelial cancer cells, regardless of CTCF status, and support an essential role of epigenetic reprogramming during cancer progression.

### Reduced CTCF Levels Lead to Loss of Insulation of SubTAD Structures.

**Fig. 6.**
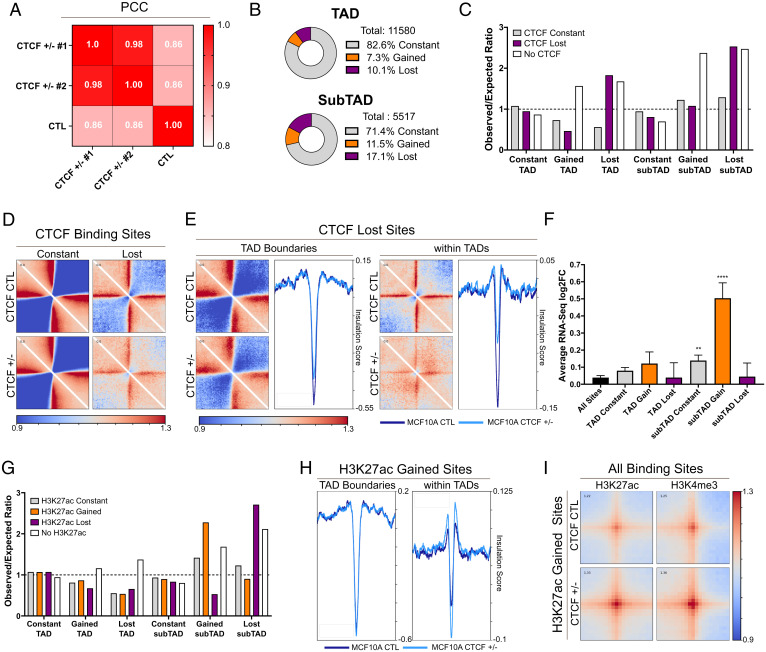
Loss of subTAD insulation drives gene expression changes. (*A*) Pearson correlation coefficient (PCC) heatmaps showing diverging contact frequencies between CTCF^+/−^ 1 and 2 and CTL. (*B*) Partitioning of constant, gained, and lost TAD and subTAD boundaries (±10 kb). (*C*) Enrichment of CTCF sites at boundaries (ratio of observed to expected (O/E)), showing an association between loss of CTCF and lost boundaries and absence of CTCF and altered boundaries. These are both more pronounced for subTAD boundaries. (*D*) Pileup plots showing local interaction, relative to randomize average genomewide interaction, around constant and lost sites of CTCF (range: 200 kb). CTCF lost sites show less insulation in CTL MCF10A, which is further reduced upon loss of CTCF. (*E*) Pileup plots of local interactions at CTCF sites localizing at TAD boundaries or within TADs. The profile plot of the average insulation score in each region quantifies the specific loss of insulation observed at lost sites of CTCF within TADs. (*F*) Average RNA-seq log2FC between CTCF^+/−^ and CTL of genes colocalizing with TAD and subTAD boundaries (±10 kb) (mean ± SEM), showing that gained subTAD boundaries are strongly associated with up-regulation of gene expression. (*G*) Enrichment of altered H3K27ac sites at altered subTAD, but not TAD, boundaries (O/E ratio). Dashed lined represents an O/E ratio of 1. (*H*) Increased average insulation score at sites of gained H3K27ac within TAD but not at TAD boundaries (colocalization: ±10 kb, range: 200 kb). (*I*) Pileup plots of increased interaction between gained H3K27ac and all sites of H3K27ac and H3K4me3 (range: 50 kb). See also *SI Appendix*, Fig. S5.

Following our ChIP-seq experiments, we posited that the loss of CTCF binding and the relative increase in open chromatin at activated genes may stem from a loss of insulation. Therefore, we investigated changes in 3D chromatin architecture using Hi-C. We generated 600 million reads per condition with biological replicates of the CTL, and replicates of CTCF^+/−^ 1 and 2 were merged for high-resolution analysis. This sequencing depth allowed us to reach complete genomic coverage at a 5-kb resolution, consistent with previous high-resolution Hi-C data (43, 44). Statistical analysis of the correlation between the contact matrices of each cell line revealed a marked difference between the CTL and both CTCF^+/−^ clones at 5-kb resolution (Fig. 6*A*), while all three groups were more homogenous when the resolution was moved to 500 kb or 1 Mb (*SI Appendix*, Fig. S5*A*). Consistent with this analysis, at the megabase scale, we also did not detect notable genomewide changes in chromosome organization between CTCF^+/−^ and CTL cells (*SI Appendix*, Fig. S5*B*), which were strikingly consistent with previously published data (45).

Next, we queried whether more local changes in chromatin architecture may underlie the RNA profiles resulting from CTCF CNL. First, we used a hierarchical TAD caller, hiTAD (46), to call TAD boundaries and domain boundaries within TADs (the subTADs) at a 10-kb resolution. We then compared the colocalization of called boundaries (±10 kb) between the CTL and two CTCF^+/−^ clones. Of the 11,580 TAD boundaries called, 10% were lost in both CTCF^+/−^ lines compared to the CTL. These changes were more pronounced when looking at subTAD boundaries, where 17% of the total number were lost (Fig. 6*B*). The loss of these boundaries might potentiate de novo contacts between DNA elements due to loss of insulation. Indeed, CTCF^+/−^ cells gained 810 TAD boundaries (7% gain) and 606 subTAD boundaries (11% gain), which are enriched next to lost boundaries (*SI Appendix*, Fig. S5*C*), indicating a reorganization of subgenomic regions. Altered boundaries frequently colocalized with altered sites of CTCF binding (±10 kb), with lost boundaries showing marked enrichment for lost CTCF elements while gained boundaries are generally CTCF null (Fig. 6*C*). These de novo TAD/subTAD interactions, demarcated by gained boundaries, are likely generated from a loss of insulator activity that limits long-range DNA contacts, so it is logical that these regions would be devoid of CTCF. This mechanism is supported by a recent study demonstrating that CTCF-independent enhancer looping is potentiated by the loss of proximal CTCF binding (47).

To validate that the loss of CTCF binding leads to local loss of DNA insulation, we imaged the average local interaction centered around lost sites of CTCF (±200 kb) (Fig. 6*D*). In agreement with our hypothesis, we detected a marked reduction of boundary strength, as represented by a decreased interaction intensity at CTCF sites delimiting two domains. DNA insulation was also clearly compromised, as represented by an increased interaction intensity between the domains spanning the lost sites of CTCF (Fig. 6*D*).

Subsequently, we asked whether loss of insulation was equally compromised at TAD and subTAD boundaries. To answer this question, we subdivided the lost CTCF sites into lost sites colocalizing with TAD boundaries or located within TADs. For each subset of the lost sites, we plotted local interactions centered around the lost sites of CTCF (±200 kb) and measured the average insulation score of these regions using framework of analysis of C-like data(FAN-C) (48). We detected a slight loss of boundary strength and insulation at lost sites of CTCF colocalizing with TAD boundaries. These results were expected since TAD boundaries are often bound by redundant CTCF sites and recent evidence indicates that many TADs insulate themselves from their neighbors independently of CTCF (49, 50). However, lost sites of CTCF within TADs resulted in a nearly complete loss of boundary strength and insulation, allowing interdomain DNA interactions (Fig. 6*E*). These observations validate, in a quantifiable manner, the prominent loss of insulation at subTADs under conditions of low CTCF expression. Since subTADs are localized within TADs, in a chromatin environment that promotes interactions, the resulting loss in insulation is more permissive to the formation of new, potentially oncogenic, contacts.

### Changes in SubTAD Organization Drives Epigenetic Reprogramming and Changes in Gene Expression.

We continued our Hi-C analysis to investigate whether the changes in subTAD interactions are connected to the changes in gene expression. First, we measured the average gene expression changes at altered subTAD and TAD boundaries. Genes colocalizing with the gained subTAD boundaries were the most significantly up-regulated (*P* < 0.0001) (Fig. 6*F*) compared to all genes. As expected, altered TAD boundaries were not significantly associated to transcriptional changes (Fig. 6*F*).

We found that altered subTAD interactions and changes to activating marks are both associated with changes in gene expression, so we investigated whether colocalization of H3K27ac or H3K4me3 was observed at domain boundaries. We detected strong enrichment of gained sites of H3K27ac and H3K4me3 with gained subTAD boundaries and, vice versa, with lost subTADs boundaries (Fig. 6*G* and *SI Appendix*, Fig. S5*D*). Altered TAD boundaries were not enriched for changes in either mark (Fig. 6*G* and *SI Appendix*, Fig. S5*D*), consistent with the lack of transcriptional changes in these regions. These results are validated by comparing the average changes in insulation at gained sites of H3K27ac colocalizing with TAD boundaries or within TADs (Fig. 6*H*). Gain of H3K27ac at TAD boundaries was not predictive of altered insulation, while gain of H3K27ac within TADs led to a marked gain of insulation (Fig. 6*H*), confirming the formation of de novo subTAD boundaries at these sites. Importantly, the genes found at gained H3K27ac within TADs were enriched for genes involved in mTor signaling (*SI Appendix*, Fig. S5*E*), as this pathway was among the 10 most differentially enriched pathways in gained H3K27ac compared constant H3K27ac within TADs. Then, using pileup plots, we looked at the average density of interactions between regions of gained H3K27ac and all sites of either H3K27ac or H3K4me3 (Fig. 6*I*). Considering all combinations, we detected a marked gain of interaction at loci where H3K27ac was gained in the CTCF^+/−^ cells (Fig. 6*I*). These results indicate that the reconfiguration of subTADs, specifically, allows for de novo interactions at regulatory regions enriched for gains of H3K27ac that drive the expression of oncogenic programs.

**Fig. 7.**
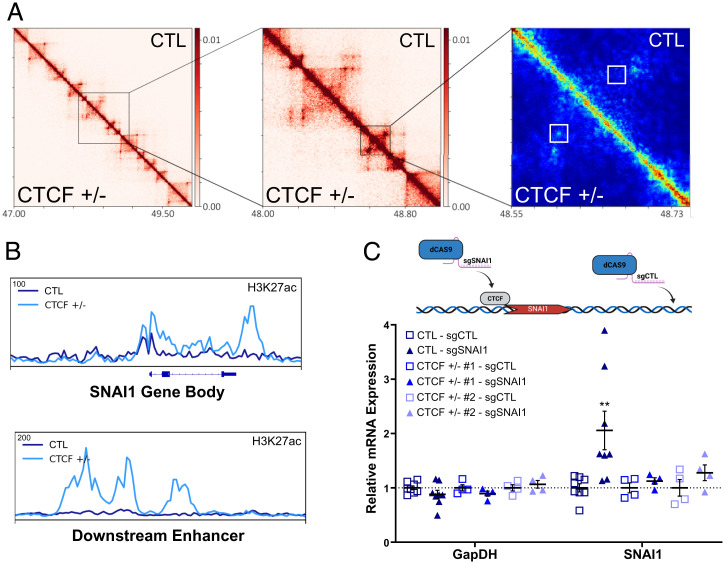
Loss of CTCF at SNAI1 drives reorganization of subTAD interactions. (*A*) Increasing zoom of the 10- and 5-kb resolution HiC heatmap to HIFI high-resolution heatmap around SNAI1 loci (chromosome 20, coordinates in megabases). Gain of enhancer-promoter interaction on the SNAI1 body, specific to CTCF^+/−^ cells, shown in the white boxes in the HIFI heatmap. (*B*) ChIP-seq track of normalized read density of increased H3K27ac on the SNAI1 gene body and the downstream enhancer, which displayed a gain of interaction in *A*. (*C*) Mean ± SEM (error bars) and individual replicates mRNA expression, relative to sgCTL, of infected CTL and CTCF^+/−^ MCF10A. SNAI1 mRNA levels (*P* = 0.0057) in CTL-sgSNAI1 compared to CTL-sgCTL. All other comparisons are nonsignificant. *Top:* Schematic of the experimental conditions. Dashed line represents a ratio of mRNA levels equal to 1, relative to controls, indicating unchanged mRNA. See also *SI Appendix*, Fig. S6.

An excellent example of this mechanism may be observed at the SNAI1 locus. At the megabase scale, conformational changes are not obvious (*SI Appendix*, Fig. S6*A*). Using Hi-C interaction frequency interference (HIFI) (51) to facilitate Hi-C resolution at a sub-5-kb scale, we detected a discrete interaction between the SNAI1 gene and a downstream potential enhancer in CTCF^+/−^ cells (Fig. 7*A*). This interaction is positioned adjacent to the lost CTCF binding site within the SNAI1 promoter and is embedded with a region of gained H3K27ac (Fig. 7*B*). The downstream enhancer, connecting with the promoter, is likewise enriched for H3K27ac in the CTCF^+/−^ cells (Fig. 7*B*).

To validate that the loss of CTCF at SNAI1 may drive its overexpression, we directed a dCAS9 construct to the CTCF site at the SNAI1 promoter (sgSNAI1) in CTL MCF10A cells, where CTCF binding is compromised in CTCF^+/−^ cells. Using ChIP-qPCR, we validated the specific displacement of CTCF at the promoter proximal CTCF binding site (CBS) (*SI Appendix*, Fig. S6*B*). Disruption of CTCF at this site with exogenous dCAS9 would be expected to facilitate an increase of SNAI1 expression if our model is correct. As CTL, we used a single guide RNA (sgRNA) targeting a CTCF-unbound region at the SNAI1 locus (sgCTL). Compared to CTL cells infected with sgCTL, cells infected with sgSNAI1 displayed a significant up-regulation of SNAI1 mRNA levels (2.1-fold increase, *P* = 0.006) (Fig. 7*C*). As a further CTL, directing dCAS9 to this CTCF binding site in CTCF^+/−^ cells, where CTCF binding is already compromised, did not result in up-regulation of SNAI1 (Fig. 7*C*). These data validate that disruption of CTCF may play a key role in driving the up-regulation of oncogenes, including SNAI1.

In summary, the loss boundaries at the subTAD level compromises insulation from de novo contacts. These de novo contacts, and the associated enrichment for H3K27ac at these regions, in turn, play a major role in driving the oncogenic networks observed in cells with CTCF CNL.

## Discussion

In this study, we demonstrated that a global loss of insulation of subTAD domains, caused by reduced pools of CTCF, may promote breast cancer progression. This loss of insulation leads to shifts in subTAD boundaries, which are strongly associated with increased deposition of the activating histone marks H3K27ac and H3K4me3 and transcriptional up-regulation of oncogenes of the PI3K pathway and SNAI1. Based on the pathway’s deregulated and invasive phenotype, we posit that CTCF CNL plays a more important role in tumor progression within breast tissue than tumor initiation. This tumor-suppressive role may be context specific. Kemp et al. (15) found that CTCF heterozygous mice are prone to developing lymphomas but not solid tumor initiation. In benign ductal carcinoma in situ, clones carrying a loss of the 16q22.1 locus was observed within an heterogenous mix, and it was unclear which oncogenic hits were required for initiation of these early-stage tumors (52). However, as these tumors evolved toward an invasive phenotype, clones carrying 16q22.1 deletion were among those enriched, supporting a tumor progression role for 16q22.1 deletion at this locus.

In lymphomas, a block of differentiation must be achieved, a process distinct from the evolution of normal epithelium to cancer tissue. However, Down syndrome–related megakaryoblastic leukemia appears to reveal a role for dysfunctional CTCF in tumor progression within the hematopoietic compartment (17). Of these patients, 20% harbor CTCF mutations. GATA1 mutation is generally required for tumor initiation in megakaryoblastic leukemia, as defined by a transient loss of proliferative control. CTCF mutations, when layered onto GATA1 mutation, potentiate evolution into clinical leukemia, defined by unlimited replicative potential and impaired ability to differentiate (17). In our precancerous breast epithelial model, proliferation and morphology remain mostly unaltered, as MCF10As were not transformed by the single allele deletion of CTCF. While common pathways may be altered upon CTCF loss in both lymphocytes and ductal epithelium of the breast, the impact may be quite distinct given the tissue-specific transcriptomic profiles already in place and unique pathways required for transformation in each tissue.

As well as advancing our knowledge on the physiological importance of proper topological organization of chromatin within TADs, our data also indicate that the development of a therapeutic approach aimed at restoration of insulation at specific genomic sites could prove useful to prevent metastasis. While such an approach may be challenging, we suggest that tumors defined by reorganization of long-range contacts may be susceptible to mTor inhibitors and HATi.

Most TAD boundaries are often bound by multiple, redundant CTCF binding sites and colocalize with transfer RNAs (tRNA) or housekeeping genes that are constitutively active (53). Unlike TADs, which are generally consistent across tissue types, subTADs reorganize themselves to allow for dynamic and precise transcriptional control of genomic loci (54). Consistent with this concept, long-range chromatin interactions are reorganized during serum starvation, dependent upon interactions between CTCF and binding partners (55), and a gain of CTCF-mediated interactions at the subTAD level has been correlated with gene expression (56). While the transcriptional regulation of oncogenic signaling pathways is not fully understood, we now show that reorganization of subTADs may play an important role in the control of oncogenic signaling. PI3K signaling is influenced by broad biological processes, including signal transduction emanating from growth factor receptors, oxidative stress, and nutrient availability. As such, it would be logical to expect that transcriptional control of PI3K signaling would be likewise impacted by regulatory mechanisms that respond quickly to similar environmental influences. Dynamic remodeling of subTAD organization represents one such mechanism.

The histone modification H3K27ac is commonly used to demarcate contact between transcriptionally active regions of the genome (57). However, it remains unclear how chromatin contacts and histone modifications influence each other. Our data provide insight into the relationship between altered subTAD distribution and epigenetic changes. Since sites of compromised CTCF binding are generally not proximal to sites of gained H3K27ac or H3K4me3, it is unlikely that the loss of CTCF drives the gain of activating marks through direct mechanisms. For example, it is unlikely that compromised CTCF binding would lead to the loss of recruitment of antagonistic epigenetic writers, such as EZH2, at sites many kilobases away, where H3K27ac is subsequently accumulated. Therefore, we can infer that the reshuffling of subTADs is driving the redistribution of H3K27ac and H3K4me3 more so than altered CTCF itself by allowing new chromatin contacts between genomic regions. In turn, these contacts may promote the recruitment of activating chromatin writers (58, 59), leading to a reprogrammed epigenetic landscape and transcriptional changes.

Our work also highlights the potential importance of histone acetylation in the oncogenic process of invasion. Multiple independent studies have revealed an important role of histone acetyltransferases in multiple steps of cancer progression, including invasion (60, 61). Further, other independent studies associated the gain of histone acetylation and enhancer activity, driven by transcription factors (62) or other epigenetic regulators such as noncoding RNAs (63), to invasiveness. Therefore, epigenetic reprogramming events, potentially involving subTAD reorganization, might invariably underlie aggressive tumor behavior. The reprogramming of the histone acetylation landscape and enhancer activity may be observed independently of altered CTCF functions, but the loss of insulation provided by aberrant CTCF binding potentiates a topological reorganization of the chromatin, leading to enhanced de novo histone acetylation and enhancer activity. Therefore, our observations hint at the potential therapeutic effectiveness of HATi to block the epigenetic shifts necessary for tumor progression, which could be more effective in CTCF-deficient cancers.

Overall, our study highlights the interplay between long-range chromatin contacts and epigenetic remodeling and underscores the importance of defining epigenetic reprogramming in cancer as a means to uncover new therapeutic avenues.

## Methods

### Cell Culture.

For MCF10A, MDA-MB-231, MCF7, and HEK293T cell culture conditions and PDX cell line establishments and culture conditions, see *SI Appendix*, *Materials and Methods*.

### CRISPR-Cas9 KO

CTCF^+/−^ MCF10A cell lines were previously used in Hilmi et al. (21). CRISPR-Cas9 KO of CDH1 in MCF10A was performed as in Hilmi et al. (21). See *SI Appendix*, *Materials and Methods*.

### Western Blot.

Western blots were carried out as previously described (21). For western blot conducted on PDX tumors, tissue was harvested as in Savage et al. (64). See *SI Appendix*, *Materials and Methods* for details.

### Growth Curves.

Using a 12-well dish (Fisher Scientific, 3513), 15,000 cells were plated per well. Cells were fixed at days 1, 3, and 5 with 4% formaldehyde and stored at 4 °C. Cells were stained with 1 mL of a crystal violet solution (1% crystal violet, 10% ethanol) and dried. The stained cells were then diluted in 10% acetic acid. The growth ratio of cells was calculated by reading and comparing the optical density at 595 nm, the intensity of the violet dye, using a PerkinElmer multimode plate reader.

### Lentiviral Infection for CTCF Addback, SNAI1 Knockdown, and dCAS9.

CTCF addback (Genecopoeia, EX-Z8806-Lv120), SNAI1 shRNA knockdown (Sigma, NM_005985; target sequence: GCAAATACTGCAACAAGGAAT), and green fluorescent protein control vectors (Genecopoeia, EX-EGFP-Lv120) were done using lentiviral vectors packages in HEK293T cells, as described previously (21). See *SI Appendix*, *Materials and Methods* for details.

### Transfection for shRNA CTCF Knockdown.

Transfection is done following the Lipofectamine 3000 transfection reagent protocol (Invitrogen, L3000001) using 5 μg of shCTL or shCTCF plasmid from Origene (TL313675, locus ID 10664). Culture medium of 50% confluent cells was changed to Opti-MEM (Gibco, 11058-021) 30 min before transfection. Then, 6 h after transfection, culture medium was changed to normal culture medium, with 1 μg/mL puromycin. Next, 24 h before starvation, culture medium was changed to puromycin-free medium.

### Transwell Invasion Assay and Quantification.

In nonsupplemented media, cells at 70% confluencewere starved for 24 h. Then, 50,000 to 200,000 cells were seeded on an insert (Falcon, 353182) coated with 20 to 25 μg/mL Matrigel (Corning, 354230) diluted in a 0.01 M Tris and 0.7 M NaCl solution. The cells were maintained in nonsupplemented media in the insert. The inserts were then placed in companion plate chambers (Falcon, 353503) containing supplemented media used for cell culture overnight or 24 h. Then, the inserts were washed in phosphate buffered saline, fixed in 5% glutaraldehyde for 10 min, stained with a crystal violet solution (1% crystal violet, 10% EtOH) for 30 min, rinsed in water, and dried. For each biological replicate, two to three inserts were plated; for each insert, five pictures were taken at 10X resolution. The total number of invading cells per photo were counted using ImageJ software, and the average number of invasive cells per five pictures per insert were averaged within each sample and compared between samples. See *SI Appendix*, *Materials and Methods* for details.

### Mammosphere Assay, Immunofluorescence, and Quantification.

Next, 5,000 cells were seeded on a 50-μL Matrigel cushion (10 to 12 mg/mL, Corning, 354230) and maintained in supplemented media containing 4% Matrigel for 8 days. The media was carefully replaced every 3 days. Average mammosphere size was measured from bright-field microscopy images on ImageJ software. p-S6 immunofluorescence was performed using p-S6 S240/244 antibody from Cell Signaling (Rabbit, 2215S) and Goat anti-Rabbit immunoglobulin G (IgG) with Alexa 488 fluorophore (Invitrogen, A32731). DAPI was used for DNA fluorescence of the whole mammosphere used for normalization of p-S6 fluorescence quantification and mammosphere filling quantification. See *SI Appendix*, *Materials and Methods* for quantification details.

### RNA-Seq.

Total RNA was extracted according to the Sigma RNA Extraction Kit (Sigma, RTN350-1KT) protocol. RNA was sent to Genome Quebec for poly(A) RNA library preparation using the NEBNext Ultra II Directional RNA Library Prep Kit for Illumina and sequencing of 50 M 100-bp paired-end reads per replicate on the Illumina NovaSEq 6000 platform. See *SI Appendix*, *Materials and Methods* for detailed data processing, analysis, full GSEA gene pathway names, and the quantitative real-time PCR protocol.

### TCGA Data Analysis.

The RNA-seq data of breast cancer patients from the TCGA dataset (generated by the TCGA Research Network: https://www.cancer.gov/tcga) were used. See *SI Appendix*, *Materials and Methods*.

### ChIP-Seq.

Minimally, 15 ng of each ChIP sample, with two biological replicates and one input per cell line (besides the ChIP-seq on CTCF in CTL MCF10A, where four biological replicates were generated), was sent to Genome Quebec (for CTCF and H3K4me3) or the Centre for Applied Genomics (TCAG) at SickKids Hospital (for H3K27ac) for library preparation using the NEB Ultra II DNA kit (no shearing) and next-generation sequencing on the Illumina platform (single-end 100-bp sequencing, ∼50 M reads per sample). See *SI Appendix*, *Materials and Methods* for ChIP-seq preparation and analysis details and the ChIP-qPCR protocol.

### EPIC Array Investigation.

Bisulfite conversion was performed using the EZ DNA Methylation Kit (Zymo Research, D5001). 500 ng per sample of bisulfite-converted DNA was sent to the Princess Margaret Genomics Centre for quality control and detection of methylated bases using the Illumina human methylation EPIC array. The IDAT files outputted from the Illumina EPIC array experiment were analyzed using the Minfi package (65) for comparison of individual red/green CpG probe intensity and genomic annotation, using the Illumina methylation EPIC reference ilm10b4.hg19. Quality control of the methylation pattern was performed using the Shinymethyl R package (66). See *SI Appendix*, *Materials and Methods* for methyl array data analysis.

### Hi-C.

Hi-C data were generated from 1 M CTL, CTCF^+/−^ 1, or CTCF^+/−^ 2 MCF10A cells per replicate, with two biological replicates per condition, using the Arima-HiC kit, according to the manufacturer’s protocols (Arima Genomics). Library preparation was performed using the KAPA Hyper Prep Kit (07962312001) following the Arima protocol for library preparation. Libraries were sent at TCAG at SickKids Hospital for next-generation sequencing using the Illumina NovaSeq S1 flow cell (paired-end 50-bp sequencing, ∼30 0 M reads per replicate). See *SI Appendix*, *Materials and Methods* for Hi-C analysis details.

### Quantification and Statistical Analysis.

Unless stated otherwise, all graphical representations display the mean and SEM of the sample’s distribution. Unless stated otherwise, graphics and statistical tests were generated and performed using GraphPad Prism 9.1 (GraphPad Software, San Diego, CA). Unless stated otherwise, all Student’s *t* tests used the one-tailed method. Graphical models were created using BioRender.

## Supplementary Material

Supplementary File

## Data Availability

All next-generation sequencing data are available at the Gene Expression Omnibus (GEO) (accession no. GSE183381) (67). All other study data are included in the article and/or *SI Appendix*.
