# Supplementary material for: 3D chromatin remodeling potentiates transcriptional programs driving cell invasion
Source: Proc Natl Acad Sci U S A. 2022 Aug 29;119(36):e2203452119. doi: 10.1073/pnas.2203452119 (PMC9457068; doi:10.1073/pnas.2203452119)
Supplement: Supplementary File [file pnas.2203452119.sapp.pdf]

Supplementary Information

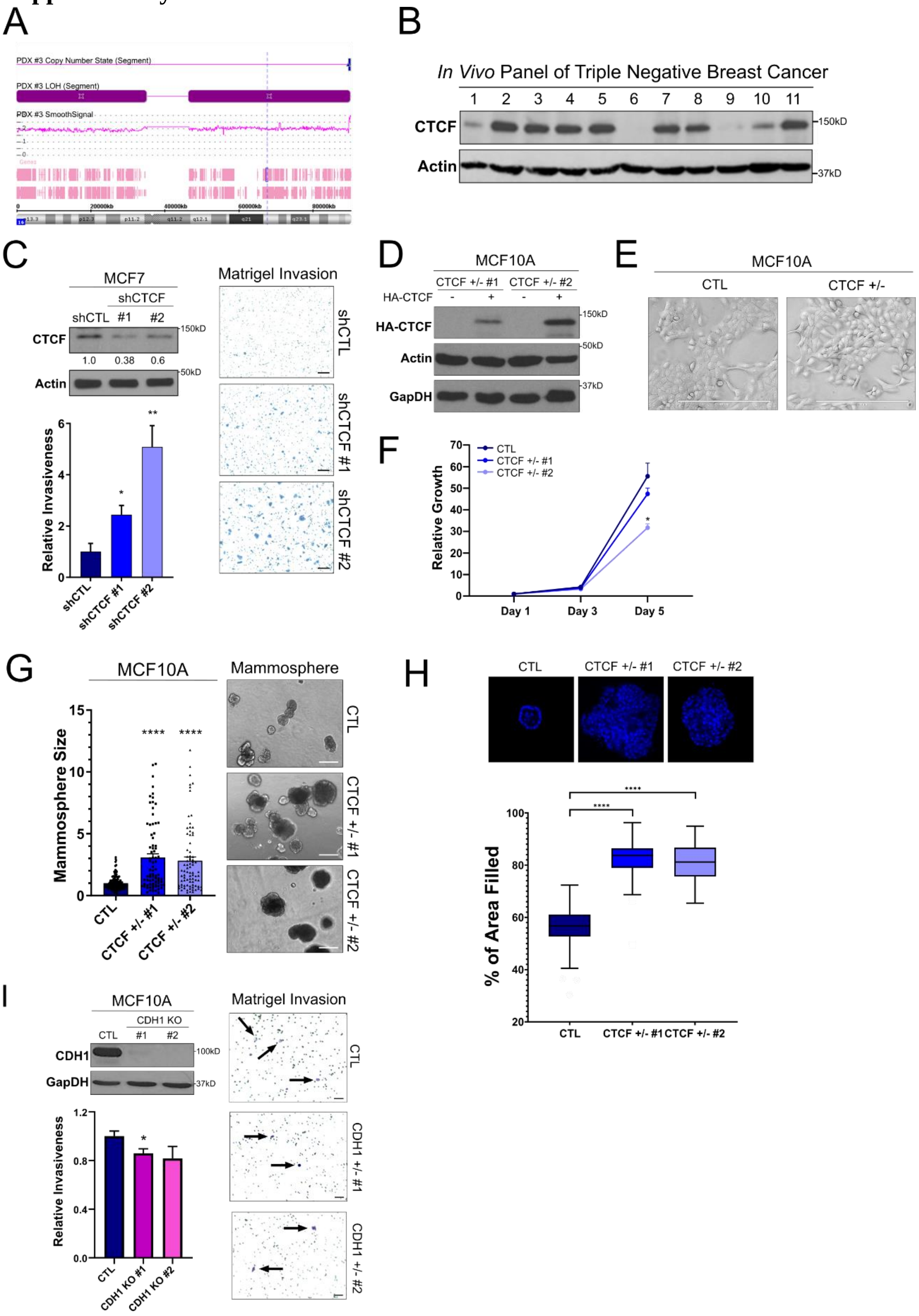

Supplementary Fig. 1.

(**A**) Copy number analysis of PDX #3 cell line using the Chromosome Analysis Suite from Thermo Fisher showing CTCF Loss of Heterozygosity (demarcated by a purple box) at the CTCF loci (demarcated by the dotted vertical line). (**B**) Western Blot of tumors from triple negative breast cancer patient derived xenograft showing that physiological CTCF levels found in tumors 1, 9 and 10 are similar to the CTCF level of our cell line models. (**C**) Western Blot of CTCF levels following shCTCF expression in MCF7 with quantification of relative CTCF band intensity to shCTL. Loading control: Actin. Bar chart of relative invasiveness of MCF7 shCTCF #1 and #2 to shCTL (mean ± SEM). $p = 0.021$ and 0.0051 for shCTCF #1 and #2 compared to shCTL, showing an increased invasiveness following shCTCF treatment. (**D**) Western Blot showing ectopic expression of HA-CTCF in CTCF+/- cells. Actin and GapDH are used as loading control. (**E**) Brightfield microscopy picture of CTL and CTCF+/- MCF10A at similar confluence, showing no major morphological changes between the cell lines. (**F**) Line chart (mean ± SEM) of the growth of CTL, CTCF+/- #1 and CTCF+/- #2 MCF10A during a 5-day growth curve assay. CTCF+/- #2 proliferates at a modestly slower rate than CTL ($p = 0.02$ using a two-tailed Student's T Test). All other comparisons are non-significant. (**G**) Average and individual mammosphere size of CTCF+/- MCF10A relative to CTL (mean ± SEM) showing an increase in mammosphere size in CTCF +/- cells, p-values < 0.0001 for both CTCF+/- #1 and #2. All p-values were calculated using Student's T Test. (**H**) Mammosphere DAPI immunofluorescence of CTL and CTCF+/- MCF10A showing an increased filing of the CTCF+/- mammospheres. Tukey Box plot below images represents the distribution of mammosphere filling in each cell lines, showing a significant increased in cells filling the core of CTCF+/- MCF10A mammospheres compared to CTL. $p < 0.0001$ for CTCF+/- #1 and #2 using a two-tailed Student's T Test. (**I**) Western blot of CDH1 levels. GapDH was used as a loading control. Bar chart (mean ± SEM) below represents the relative invasiveness in a matrigel invasion assay of each cell line, normalized to CTL, showing no increase in invasiveness following CDH1 KO. $p = 0.0459$ and 0.129 for CDH1 KO #1 and #2 compared to CTL using a one-tailed Student's T Test. Pictures on the right show the inserts following invasion. The few invading cells are indicated with black arrows.

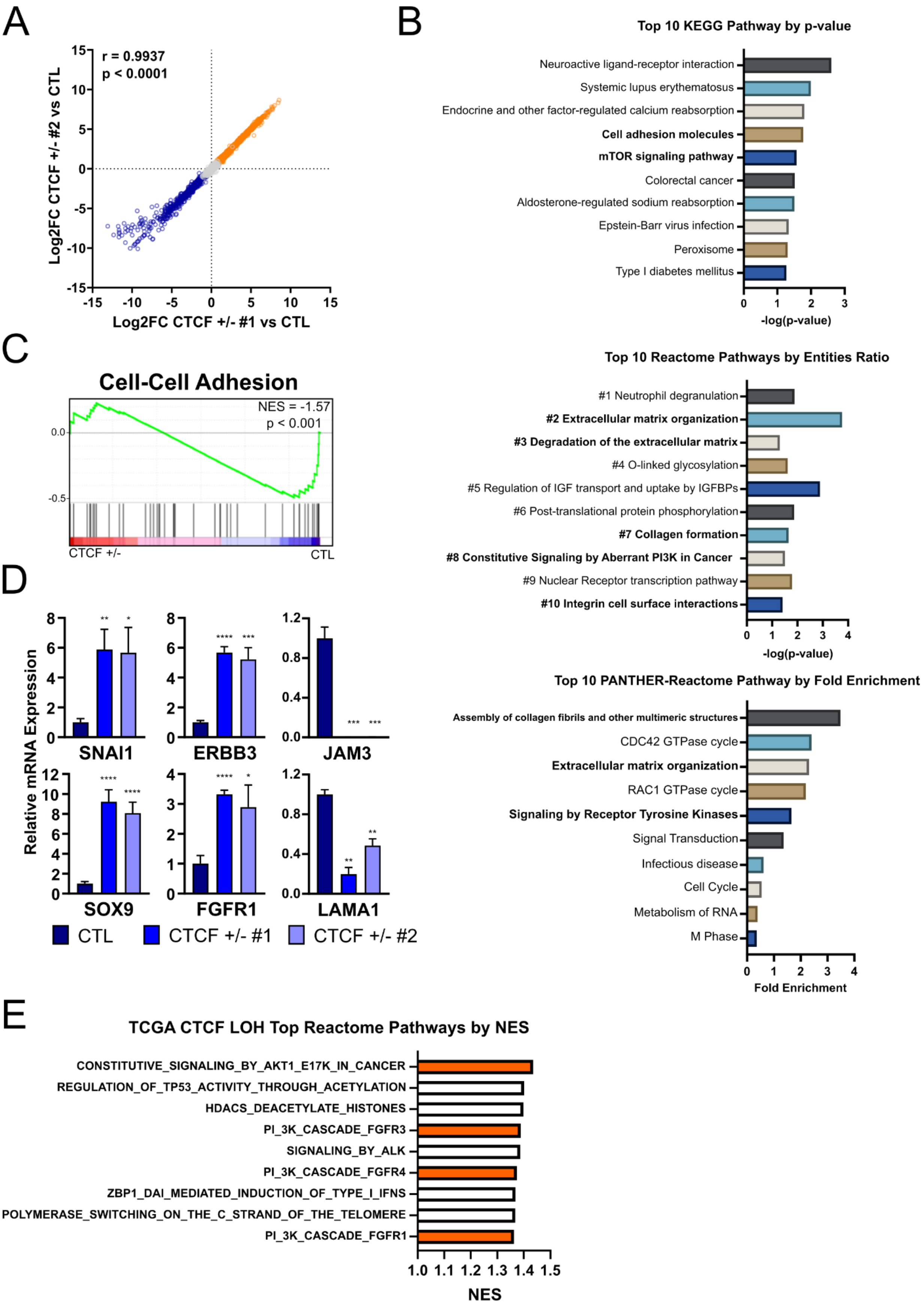
A
r = 0.9937
p < 0.0001
Log2FC CTCF +/- #2 vs CTL
Log2FC CTCF +/- #1 vs CTL
B
Top 10 KEGG Pathway by p-value
Neuroactive ligand-receptor interaction
Systemic lupus erythematosus
Endocrine and other factor-regulated calcium reabsorption
Cell adhesion molecules
mTOR signaling pathway
Colorectal cancer
Aldosterone-regulated sodium reabsorption
Epstein-Barr virus infection
Peroxisome
Type I diabetes mellitus
-log(p-value)
Top 10 Reactome Pathways by Entities Ratio
#1 Neutrophil degranulation
#2 Extracellular matrix organization
#3 Degradation of the extracellular matrix
#4 O-linked glycosylation
#5 Regulation of IGF transport and uptake by IGFBPs
#6 Post-translational protein phosphorylation
#7 Collagen formation
#8 Constitutive Signaling by Aberrant PI3K in Cancer
#9 Nuclear Receptor transcription pathway
#10 Integrin cell surface interactions
-log(p-value)
Top 10 PANTHER-Reactome Pathway by Fold Enrichment
Assembly of collagen fibrils and other multimeric structures
CDC42 GTPase cycle
Extracellular matrix organization
RAC1 GTPase cycle
Signaling by Receptor Tyrosine Kinases
Signal Transduction
Infectious disease
Cell Cycle
Metabolism of RNA
M Phase
Fold Enrichment
C
Cell-Cell Adhesion
NES = -1.57
p < 0.001
CTCF +/-
CTL
D
Relative mRNA Expression
SNAI1
ERBB3
JAM3
SOX9
FGFR1
LAMA1
CTL
CTCF +/- #1
CTCF +/- #2
E
TCGA CTCF LOH Top Reactome Pathways by NES
CONSTITUTIVE_SIGNALING_BY_AKT1_E17K_IN_CANCER
REGULATION_OF_TP53_ACTIVITY_THROUGH_ACETYLATION
HDACS_DEACETYLATE_HISTONES
PI_3K_CASCADE_FGFR3
SIGNALING_BY_ALK
PI_3K_CASCADE_FGFR4
ZBP1_DAI_MEDIATED_INDUCTION_OF_TYPE_I_IFNS
POLYMERASE_SWITCHING_ON_THE_C_STRAND_OF_THE_TELOMERE
PI_3K_CASCADE_FGFR1
NES

Supplementary Fig. 2.

(**A**) Correlation of changes in gene expression of the respective CTCF +/- clones to CTL (Pearson r and p-value), showing a strong reproducibility of gene expression changes in both cell lines. (**B**) Top 10 KEGG and Reactome pathway analysis by p-value and entities ratio, respectively, for upregulated genes (Log2FC >= 2, adjusted p-value <= 0.05). Top 10 Reactome pathways, analysed with PANTHER and ranked by fold enrichment, of significantly up or downregulated genes (abs(Log2FC) >= 1, adjusted p-value <= 0.05). Gene sets related to PI3K signaling pathway or EMT are written in bold and are distinctly present in the top 10 of each analysis method. (**C**) GSEA Analysis highlighting the downregulation of cell-cell adhesion pathway from the Gene Ontology data set. (**D**) Bar chart (mean ± SEM) of the qPCR validation of the top hits in the RNA-Seq, showing the relative expression normalized on 3 housekeeping genes compared to CTL. p-values are listed below and were calculated using a two-tailed Student's T Test comparing CTCF+/- #1 and #2 to CTL: SNAI1 #1 = 0.0075 and #2 = 0.0175 ; ERBB3 #1 < 0.001 and #2 = 0.0004 ; SOX9 #1 and #2 < 0.0001 ; FGFR1 #1 < 0.0001 and #2 = 0.0379; JAM3 #1 and #2 = 0.0001 ; LAMA1 #1 = 0.0016 and #2 = 0.0081. (**E**) Top 10 Reactome Pathways ranked by NES of the GSEA Prerank analysis on TCGA dataset as described in Figure 2C. Pathways related to PI3K signaling are highlighted in orange and dominate the top 10.

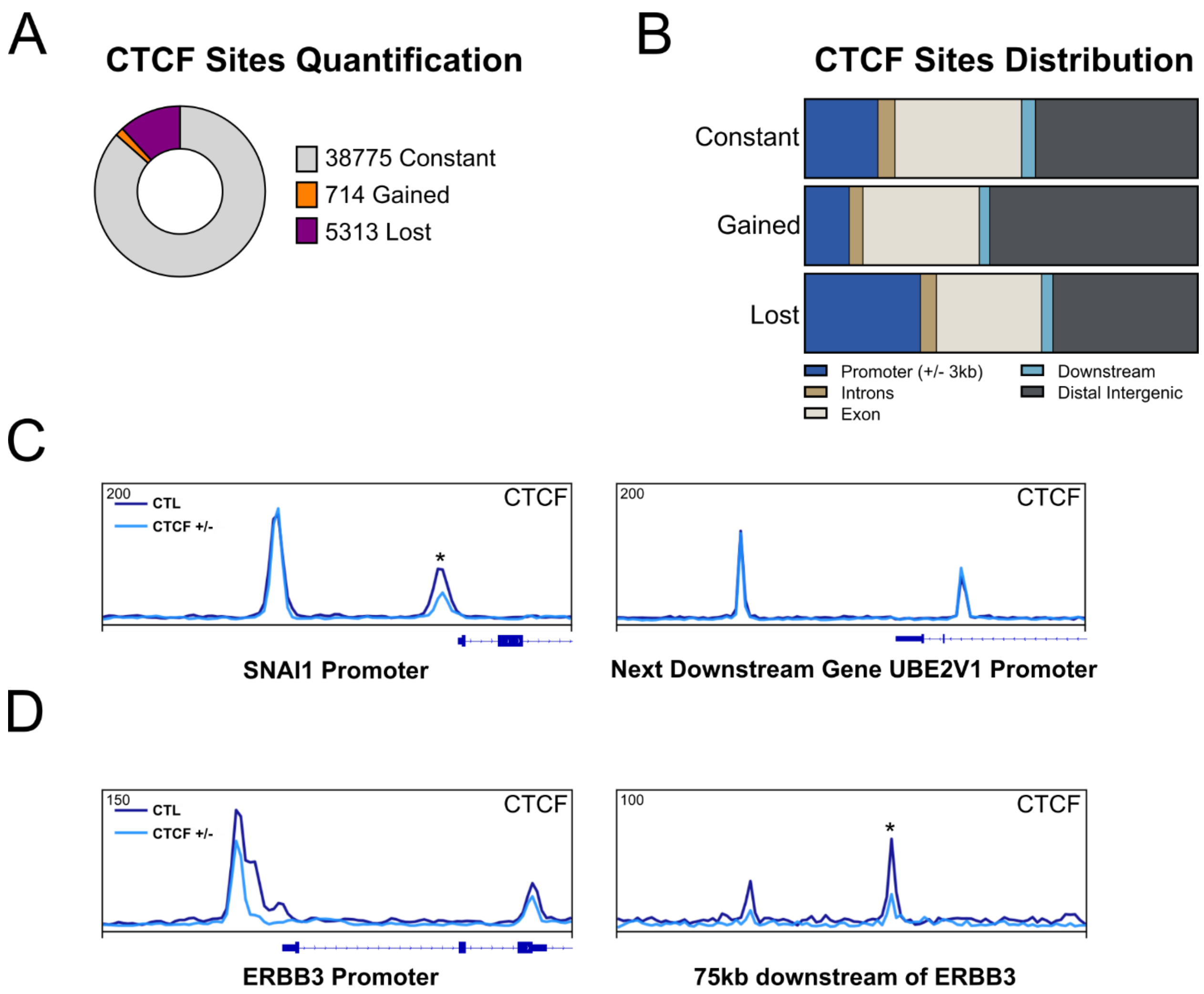

Supplementary Fig. 3.

(**A**) Partitioning of constant lost and gain sites from Figure 4A. (**B**) Genomic distribution of the different clusters, shows a small enrichment of lost sites on promoters and gain sites on distal intergenic sites, compared to constant CTCF binding sites. (**C**/**D**) ChIP-Seq track of normalized read density showing the specific loss of CTCF binding at SNAI1 promoter and downstream of ERRB3 ($p <= 0.05$ calculated using DiffBind 3.0), as ChIP-Seq track of surrounding regions show no significant change in CTCF binding. The normalized read density of each track ranges from 0 to the number noted in the top left corner.

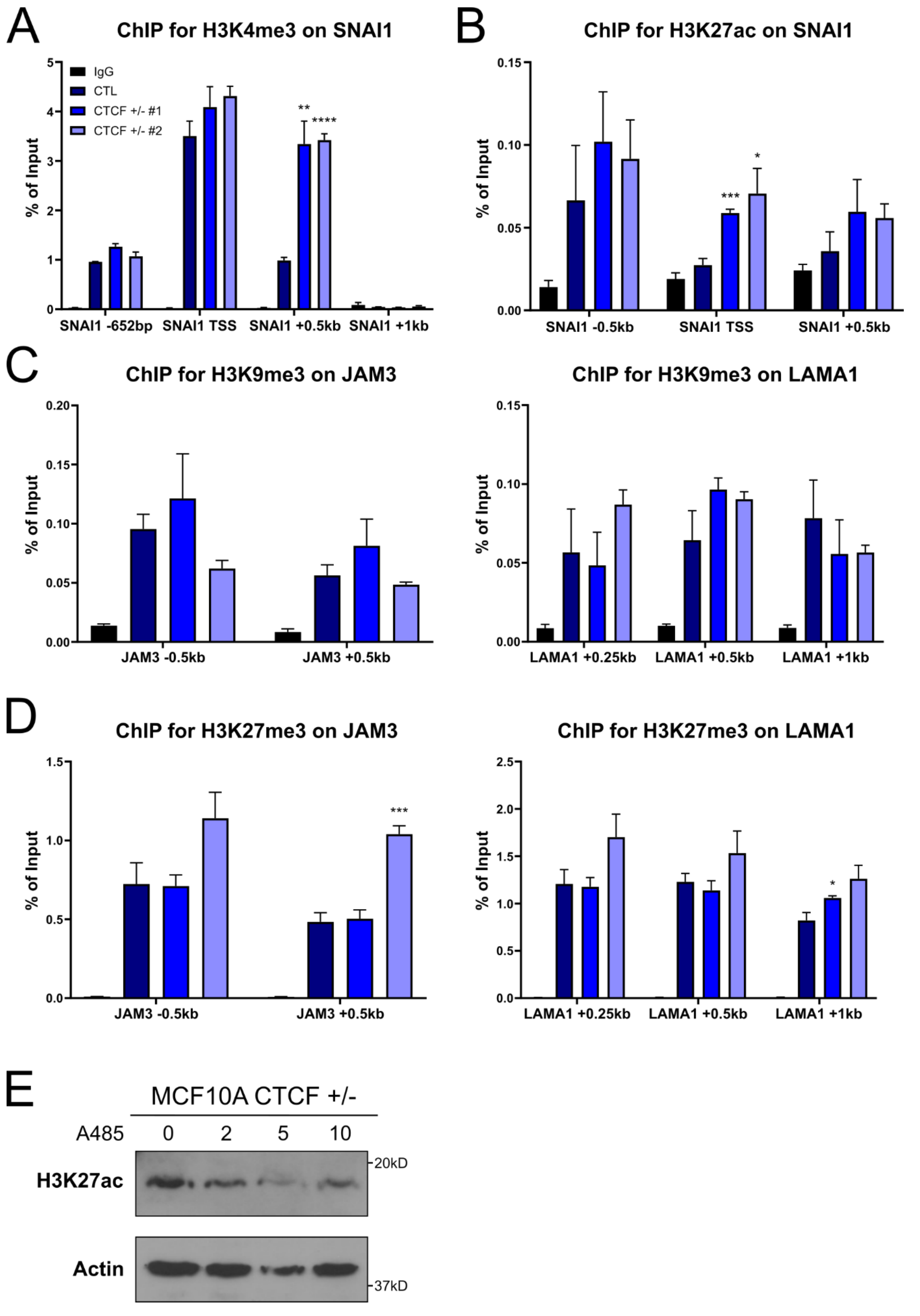

A
ChIP for H3K4me3 on SNAI1
IgG
CTL
CTCF +/- #1
CTCF +/- #2
% of Input
**
****
SNAI1 -652bp
SNAI1 TSS
SNAI1 +0.5kb
SNAI1 +1kb
B
ChIP for H3K27ac on SNAI1
% of Input
***
*
SNAI1 -0.5kb
SNAI1 TSS
SNAI1 +0.5kb
C
ChIP for H3K9me3 on JAM3
% of Input
JAM3 -0.5kb
JAM3 +0.5kb
ChIP for H3K9me3 on LAMA1
% of Input
LAMA1 +0.25kb
LAMA1 +0.5kb
LAMA1 +1kb
D
ChIP for H3K27me3 on JAM3
% of Input
***
JAM3 -0.5kb
JAM3 +0.5kb
ChIP for H3K27me3 on LAMA1
% of Input
*
LAMA1 +0.25kb
LAMA1 +0.5kb
LAMA1 +1kb
E
MCF10A CTCF +/-
A485 0 2 5 10
H3K27ac
20kD
Actin
37kD

Supplementary Fig. 4.

ChIP-qPCR Screening of histone modifications around genes with altered expression in CTCF+/- MCF10A. All bar charts represent mean % of input ± SEM. The coordinates of each site is represented as distance to TSS. (**A**) ChIP-qPCR results showing the significant gain of H3K4me3 in CTCF+/- MCF10As relative to CTL at 500bp downstream of SNAI1. $p = 0.00729$ and 0.000068 in CTCF+/- #1 and #2, respectively. (**B**) ChIP-qPCR results showing the significant gain of H3K27ac in CTCF+/- MCF10As relative to CTL SNAI1 TSS. $p = 0.00169$ and 0.0247 in CTCF+/- #1 and #2 respectively. (**C**) ChIP-qPCR results showing no significant changes in H3K9me3 binding between CTCF+/- and CTL MCF10As around JAM3 and LAMA1 TSS. (**D**) ChIP-qPCR results showing no consistent significant changes in H3K27me3 binding between CTCF+/- and CTL MCF10As around JAM3 and LAMA1 TSS. P-values at JAM3 TSS +0.5kb = 0.806 and 0.000170; p-values at LAMA1 TSS +1kb =0.0143 and 0.0708 in CTCF+/- #1 and #2, respectively. (**E**) Western Blot showing the decrease in H3K27ac levels following A485 treatments (in µM) in MCF10A CTCF+/- cell.

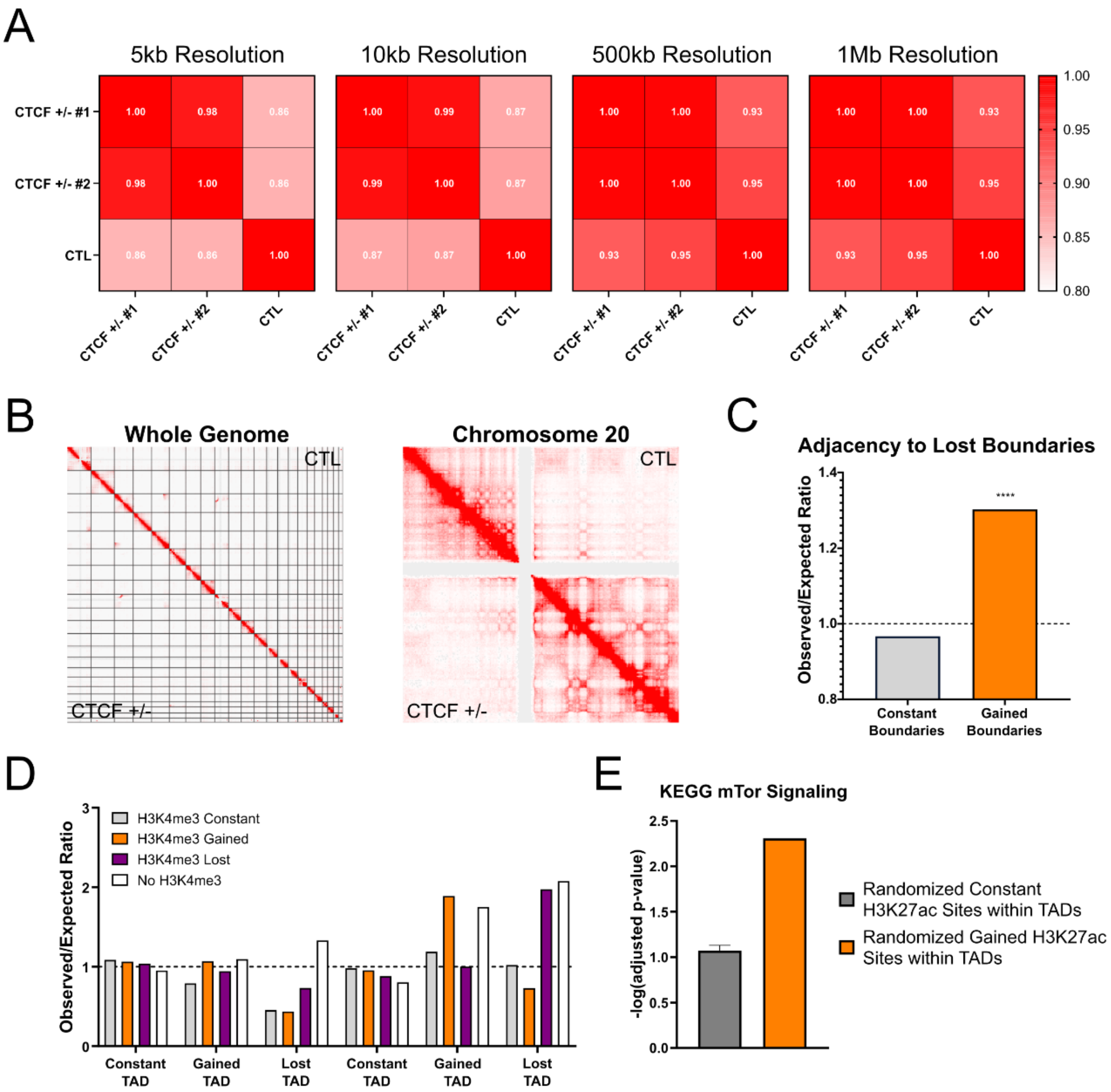
A
5kb Resolution
10kb Resolution
500kb Resolution
1Mb Resolution
CTCF +/- #1
CTCF +/- #2
CTL
1.00
0.95
0.90
0.85
0.80
B
Whole Genome
Chromosome 20
CTL
CTCF +/-
C
Adjacency to Lost Boundaries
Observed/Expected Ratio
****
Constant Boundaries
Gained Boundaries
D
H3K4me3 Constant
H3K4me3 Gained
H3K4me3 Lost
No H3K4me3
Observed/Expected Ratio
Constant TAD
Gained TAD
Lost TAD
E
KEGG mTor Signaling
-log(adjusted p-value)
Randomized Constant H3K27ac Sites within TADs
Randomized Gained H3K27ac Sites within TADs

Supplementary Figure 5: Hi-C Analysis Additional Figures

(**A**) Pearson Correlation Coefficient heat maps comparing the contact frequencies between CTCF +/- #1 and #2 and CTL, showing a distinction between CTL and CTCF +/- at high resolution, where shorter interactions can be surveyed, while this distinction fades at lower resolution. (**B**) Juicebox heatmap of balanced interaction read count for the whole genome and chromosome 20, where SNAI1 loci is located. Interactions in CTL MCF10A are represented in the top/right half, while interaction in CTCF+/- are represented in the bottom/left half of each heat map. (**C**) Bar Chart (Observed/Expected Ratio) representing the enrichment of constant or gained boundaries adjacent to a lost boundaries (+/- 1 boundaries) showing that gained boundaries are significantly enriched around lost boundaries (Chi Square Test, $p < 0.0001$). (**D**) Bar chart (O/E Ratio) showing the enrichment of gained H3K4me3 on gained subTAD and vice-versa for lost H3K4me3 and lost subTAD. (**E**) Differential Enrichment of mTor Signaling KEGG Pathway at gained H3K27ac sites within TADs compared to 100 equinumerous subsets of constant H3K27ac sites within TADs.

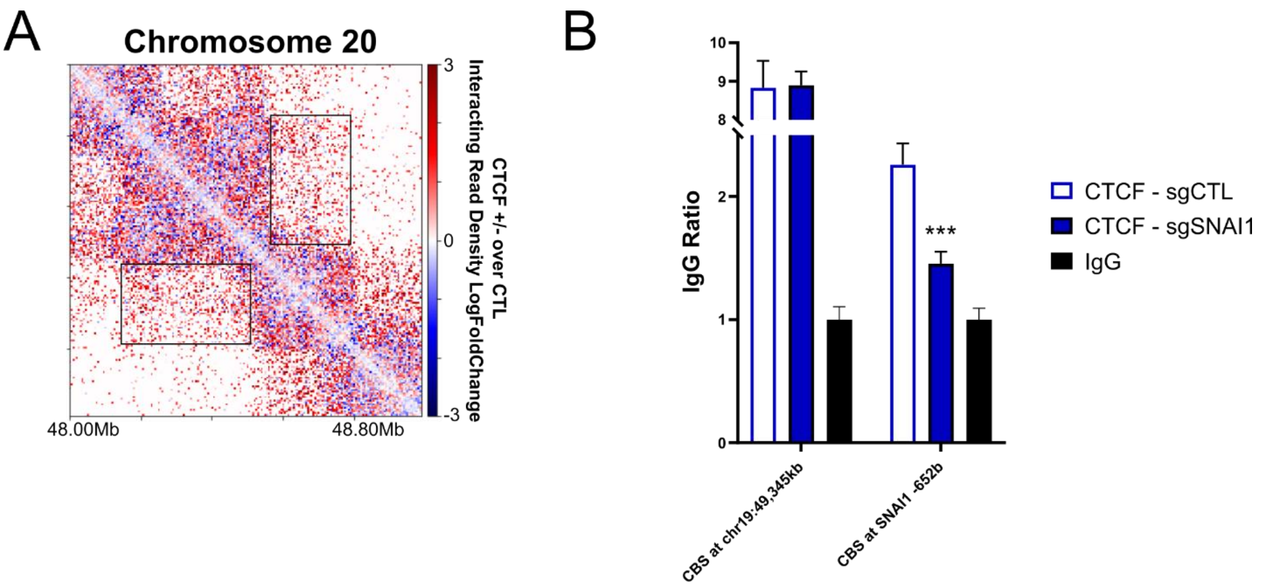

Supplementary Figure 6: Minor Loss of Insulation at the TAD Level around SNAI1
(**A**) Symmetrical comparative heat map of the differential interaction (in logFC) of CTCF+/- compared to CTL MCF10A at the same genomic region as the second heatmap in Figure 7A. The black boxes highlight a zone of modestly increased interdomain interactions in the CTCF+/- compared to CTL MCF10A. (**B**) Bar chart (IgG Ratio +/- SEM, n =4) of CTCF ChIP-qPCR results in CTL MCF10A following dCAS9 and sgCTL or sgSNAI1 expression showing a significant reduction of CTCF binding at the target site upstream of SNAI1 by sgSNAI1. As control, CBS at chr19:49,345kb is not target by sgSNAI1 and is therefore not hindered.

Appendix, Materials and Methods

Cell Culture Details:

MCF10A cell lines were maintained in DMEM/F12 50/50 (Wisent, #319-085-CL) supplemented with EGF (100µg/ml, Wisent, #511-110-UM), Insulin (10mg/ml, Wisent, #H511-016-U6), Hydrocortisone (1mg/ml, Sigma, #H0888-1G), Horse Serum (2%, Wisent, #065150) and Choleratoxin (1mg/ml, Sigma, #C8052-2MG) in an incubator at 37C and 5% CO2.
MDA-MB-231, MCF7, SKBR3 and HEK293T cell lines were maintained in DMEM (Wisent, #319-005-CL) supplemented with 10% FBS (Gibco, #12483-020) in an incubator at 37C and 5% CO2.
Conditionally Reprogrammed Cells of Patients Derived Xenograft of Triple Negative Breast Cancer tumors, termed PDXs, were established following as in Liu et al. 2017 (1) and Sirois et al. 2019 (2) and given to us by Dr. Park's and Dr. Basik's Laboratories from McGill University. Low CTCF protein expression in these cell lines was confirmed by Western Blot. Loss of Heterozygosity was confirmed using the Chromosome Analysis Suite from Thermo Fisher. They were maintained in DMEM (Wisent, #319-005-CL), with 25% Ham's F12 (Wisent, #312-250-CL), 8% FBS (Gibco, #12483-020), L-Glutamine (1.5mM, Wisent, #609-065-EL), EGF (50µg/ml, Wisent, #511-110-UM), Insulin (5mg/ml, Wisent, #H511-016-U6), Hydrocortisone (0.8mg/ml, Sigma, #H0888-1G), Choleratoxin (84µg/ml, Sigma, #C8052-2MG), RhoK Inhibitor (0.01mM, Y-27623, StemCell Technologies, #72304) in an incubator at 37C and 5% CO2.

CRISPR-Cas9 Knockout Details:

In brief, 1 million MCF10A cells are seeded in a 6cm dish and transfected the next day using Lipofectamine 3000 (Invitrogen, # L3000001), 6µg of pCas-Guide-ef1a-GFP (OriGene, #GE100018, guide sequence : ACGTCGTTACGAGTCACTTCAGG). Two days later, GFP-positive cells were isolated by fluorescence-activated cell sorting into 96-well plates (one cell per well). To screen for CDH1 KO clones, we isolated genomic DNA of each clone and amplified proximal sequences surrounding the Cas9 targets by polymerase chain reaction. Positive clones were first identified using the SURVEYOR Assay Kit (IDT, #706020) followed by Sanger Sequencing at Genome Quebec facilities.

Western Blot Protocol:

For all Western Blots, cells were harvested by scrapping. Then, cells are lysed in whole-cell lysis buffer [20 mM tris (pH 7.5), 420 mM NaCl, 2 mM MgCl2, 1 mM EDTA, 10% glycerol, 0.5% NP-40, 0.5% Triton X-100, supplemented with fresh 1 mM dithiothreitol, phenylmethylsulfonyl fluoride, protease inhibitor cocktail (Roche) and phosphatase inhibitors, bis-glycerol phosphate, and NaF] for 15min, then spined at 13000rpm at 4°C for 15min to pellet cellular debris. Then, the protein concentration of the supernatant is assessed using a Bradford assay (Fisher, #1856209). 40µg of proteins are loaded on an 8 to 12% acrylamide gel and electrophoresed at 120V for 1h. Then, proteins on the gel are transferred on nitrocellulose membrane (Pall, #66485) at 4°C, 34V overnight for 8% gel and 100V for 1 hour for 12% gel. The membrane is then blocked with 5% milk in TBST [20 mM Tris base, 137 mM NaCl, and 0.1% Tween 20] for 3h at 4°C. The membrane is then incubated with primary antibodies (see Supplementary Table 1) overnight at 4°C. Membranes are rinsed and washed for 10 minutes twice with TBST prior to secondary antibody incubation with goat anti-rabbit (SeraCare, #5220-0458) or anti-mouse

(SeraCare, #5450-0011) dilute 1/10000 or 1/20000 in 5% milk in TBST. Membranes are washed again 3 times for 10 minutes in TBST, then revealed using ECL (Bio-Rad, #170-5061). Band intensity quantification and normalization on background is performed using ImageJ software.

Lentiviral Infection for CTCF Addback and SNAI1 Knockdown Details:

HEK293T cells were transfected with 7µg of the required lentiviral vector combined with 5µg of packaging vector MD2G and 2µg of envelope vector Pax2 using polyethylenimine (1 mg/ml). 24h after transfection, media was changed for the culture media used for maintenance. Viruses were collected at 48h and 72h after transfection and passed through a 0.45-µm filter. For the infection, MCF10A and PDXs cells were infected in six-well dishes and incubated with 1ml of viral supernatant along with 1ml of their respective culture media and 8µg/ml of hexadimethrine bromide (Polybrene) for MCF10A and 60µg/ml for PDXs. 24h after infection, culture media is changed and puromycin selection starts.
MCF10A cells were selected with 1 µg/ml of puromycin for the first two days following infection, followed by 0.25µg/ml of puromycin for 2 to 3 more days. Culture media is changed for puromycin free media 24h before the starvation period preceding the invasion assay. For the PDX cells, 1µg/ml of puromycin is used on the first day of selection, 0.5µg/ml of puromycin is used for the second day, culture media is changed for puromycin-free media on the third day and the preliminary starvation starts on the fourth day.

Transwell Invasion Assay and Quantification Details:

Conditions for invasion were optimized for each cell lines as follows. 70% confluent cells were starved for 24h in non supplemented DMEM/F12, for the MCF10A cells, or DMEM for MDA-MB-231, MCF7, SKBR3 and PDXs. 50 000 MCF10A cells or 100 000 MDA-MB-231 or SKBR3 cells or 200 000 PDX or MCF7 cells are seeded into an insert (Falcon, #353182) coated with 25µg/ml matrigel (Corning, #354230) for the MCF10A, MCF7 and MDA-MB-231 or 20µg/ml matrigel for the PDXs or SKBR3, diluted in in a 0.01M Tris and 0.7M NaCl solution. The cells are maintained in non supplemented media in the insert. The inserts are then placed in companion plate chambers (Falcon, #353503) containing supplemented media used for cell culture overnight for the MCF10A and MDA-MB-231 or for 24h for the PDX, MCF7 and SKBR3.

For Torin1 (Tocris, #4247) or A485 (Tocris, #6387) treated conditions, cells were treated with the indicated concentration of Torin1 (0nM and 25nM) or A485 (0µM, 2µM and 5µM), diluted in DMSO, for 24h before starvation, during starvation and during invasion, in both the insert media and the companion plate media.

Lentiviral Infection for dCAS9 Details:

20k MCF10A cells are plated in each well of a 6 well-plate. To increase the rate of infection, cells are infected sequentially over a period of 4 days: 24h and 72h after seeding, cells are infected with dCAS9+blasticidin resistance lentiviral construct (Addgene, #85417), with a ratio of media to dCAS9 viral media of 1:1 and 30ug/ul of polybrene; 48h and 96h after seeding, cells are infected with guideRNAbackbone+puromycin resistance lentiviral construct (Addgene, #52963, in which gRNA sequence (CACCGGAGGACAGAGAGACAAGTGT) generated with CHOPCHOP(3) were cloned into by Norclone), with a ratio of media to gRNA viral media of 1:1 and 30ug/ul of polybrene. Culture media is changed every 24h during infection period and

afterward, until cells are harvested. 2ug/ul blasticidin selection starts after 48h and is reduced to 1ug/ul after 72h, until cells are harvested. 1ug/ul puromycin selection starts after 72h and is reduced to 0.5ug/ul after 96h, until cells are harvested. Cells are harvested for RNA extraction 3 days after the last infection (7 days after seeding).

Mammosphere Immunofluorescence Quantification:

Mammosphere filling was quantified from Z-stacks of DAPI stained mammosphere images using ImageJ software. The ratio between the area of the hollow cavity and the total area of the mammosphere was measured on each Z-stacks of each mammosphere of each sample and the Z-stack with the highest ratio was selected and quantified for each mammosphere of each sample. Statistical test between the filling ratio of samples was performed using Student t-test.
The quantification of p-S6 fluorescence was performed using a custom script in ImageJ developed by Dr. Luke McCaffrey's group. In brief, mammospheres were detected by thresholding the image (Mean method) to create a whole-organoid mask. This mask was duplicated then iteratively eroded (13 times) to create an inner mask that excluded the outer layer of cells. A mask for the outer layer of cells was generated using an XOR gate applied to the whole organoid and inner mask. The mean pixel intensity (8-bit) was measured under the mask, for each whole organoid, outer, and inner regions. The mean pixel intensity of each region was then compared between the samples. Statistical test between the p-S6 outer fluorescence of each sample was performed using Student t-test.

RNA-Seq Data Processing and Analysis Details:

The overall quality of reads and sequencing was assessed before and after trimming using the FastQC package (Babraham Bioinformatics). Prior to mapping, reads were trimmed with Trimmomatics (4) using the following condition: ILLUMINACLIP:$Adapters:2:30:10:8:true, HEADCROP:4, SLIDINGWINDOW:4:30, LEADING:3, TRAILING:3, MINLEN:30. Alignment on hg19 human genome was performed with STAR 2.5.4b (5) default parameters, and converted into bam format using Samtools 1.9 (6). Differential expression analysis was generated using FeatureCounts count matrix (7) followed by DESEQ2 analysis (8), using default parameters and prefiltering, for comparison across samples.

<u>RNA-Seq Correlation and Volcano Plot</u>

Correlation and Volcano plot representation of the RNA-Seq results was generated using the DESEQ2 calculated Log2FC and -log(adjusted p-value) of the respective MCF10A CTCF+/- compared to MCF10A CTL for every gene with a basemean > 100. Genes with p-value < 0.05 were represented in grey. Genes with Log2FC > 0 were represented in orange. Genes with Log2FC < 0 were represented in purple.

<u>RNA-Seq GSEA Pathway Analysis</u>

Pathway analysis was performed using GSEA tools default setting on the read count matrix of all significantly altered genes (basemean > 100, p.value <0.05)(9). All gene sets shown were significant for both p-value (< 0.001) and FDR (<0.25). Pathway names were shortened as follows, with the full name of each pathway being:

PI3K Signaling : GO_PHOSPHATIDYLINOSITOL_3_KINASE_SIGNALING,
EMT : GO_EPITHELIAL_TO_MESENCHYMAL_TRANSITION,
EMT Regulation : GO_POSITIVE_REGULATION_OF_EPITHELIAL_CELL_MIGRATION,
PI3K Regulation : GO_POSITIVE_REGULATION_OF_PHOSPHATIDYLINOSITOL_3_KINASE_SIGNALING
Cell-Cell Adhesion :
GOBP_HETEROPHILIC_CELL_CELL_ADHESION_VIA_PLASMA_MEMBRANE_CELL_ADHESION_MOLECULES

PI3K Signaling in Cancer : REACTOME_PI3K_AKT_SIGNALING_IN_CANCER
Constitutive AKT Signaling : REACTOME_CONSTITUTIVE_SIGNALING_BY_AKT1_E17K_IN_CANCER

RNA-Seq KEGG and REACTOME Pathway Analysis:

Pathway analysis for KEGG and PANTHER-Reactome was done using PANTHER webtool (http://www.pantherdb.org/) (10). Reactome pathway analysis was done using Reactome webtool (https://reactome.org/) (11).

RNA-Seq Heatmaps

Heatmaps were generated using the Log2FC to the average normalized read counts in MCF10A CTL of the 20 genes with the highest absolute Log2FC in the PI3K Regulation and EMT Regulation genesets.

RT-qPCR Protocol:

Total RNA was extracted according to Sigma RNA Extraction Kit (Sigma, #RTN350-1KT) protocol. RNA quantity and quality was measured using Nanodrop. 500ng of RNA are used as template for Reverse-Transcriptase PCR, following the manufacturer protocol (All-In-One RT MasterMix, ABM, #G490). The cDNA is diluted 1:10 and 2µl is used for qPCR amplification, following the manufacturer protocol (GoTaq qPCR MasterMix 2X, Promega, #A600A). Relative levels of cDNA are compared between the samples using the $2^{-\Delta\Delta CT}$ formula normalized on the average level of 3 housekeeping genes (GapDH, RPL4 and RPLPO). Statistical test between normalized gene expression of each gene for each sample is performed using Student t-test. The sets of primers used for RT-qPCR are listed in Supplementary Table 2.

TCGA Data Analysis: For pathway analysis, Spearman correlation test was calculated between CTCF expression in patients and the individual expression of each gene surveyed in TCGA and with a baseMean > 100 in our RNA-Seq. Genes were then ranked by -log(p-value), in which p-values equal to zero were brought to the smallest non-zero p-value measured. The ranked list was then analyzed using GSEA PreRank analysis (9) and ranked by Normalized Enrichment Score (NES) or p-value. For the SNAI1 box plot, the dataset was separated into the high CTCF group, being the top 20% of patients in term of highest CTCF expression, and the low CTCF group, being the top 20% of patients in term of low CTCF expression. The two groups were compared for SNAI1 RNA expression using a Student's T Test and a Spearman correlation test.

ChIP-Seq Sample Preparation Details:

70-80% confluent cells were fixed 10 minutes in 4% formaldehyde and stored at -80C. The pellets were subsequently resuspended in 1ml of ChIP-buffer [0.25% NP-40, 0.25% Triton X-100, 0.25% Sodium Deoxycholate, 0.005% SDS, 50nM Tris (pH8), 100mM NaCl, 5mM EDTA, 1X PMSF, 2mM NaF, 1X P8340 Cocktail Inhibitor (Roche)] and sonicated with a probe sonicator (Fisher Scientific Sonic Dismembrator Model 500) using the following cycles: 5 cycles at 20% power, 5 cycles at 25% power, and 5 cycles at 30% power. Each cycle lasts 10 seconds, and the samples are kept on ice between each cycle to avoid overheating. Next, the samples are spun at high speed in a microcentrifuge for 30 minutes. Then, lysates are collected and protein concentration measured using the Bradford assay, as described above. Based on protein concentrations, samples are diluted to 2mg/ml proteins in ChIP-buffer and 50ul/ml of Protein G Plus-Agarose Suspension Beads (Calbiochem, IP04-1.5ML) are added for 3h to preclear. 2% of the sample is collected as input and kept at -20 °C until DNA purification. Immunoprecipitation is carried out at 4°C overnight with 1ml of sample, 60ul of beads and primary antibody (see Supplementary Table 1). The beads are then washed once with Wash1, Wash2, Wash3 [0.10%

SDS, 1% Triton X-100, 2mM EDTA, 20mM Tris (pH 8), 150/200/500mM NaCl for Wash 1,2,3 respectively], Wash LiCl [0.25M LiCl, 1% NP-40, 1% Sodium Deoxycholate, 1mM EDTA, 10mM Tris (pH8)] and twice with TE buffer [10mM Tris (pH8), 1mM EDTA]. Then, beads are resuspended in elution buffer [1% SDS, 0.1M $NaHCO_3$]. The samples are decrosslinked overnight at 65 °C. 20µg of Proteinase K (Sigma, # 39450-01-6) is added for 1h at 42 °C. Then, DNA is purified using BioBasic DNA collection column (BioBasic, #SD5005). DNA concentration was assessed via Picogreen assay (Invitrogen, #P7589).

ChIP-Seq Data Processing:

Quality control of reads and sequencing was assessed before and after trimming by FastQC (Babraham Bioinformatics). Reads were trimmed with Trimmomatics (4) using the following parameters: ILLUMINACLIP:$Adapters:2:30:10, LEADING:30, TRAILING:30, SLIDINGWINDOW:4:30, MINLEN:30. Alignment on hg19 human genome was performed using BWA (6) default conditions. Sam files generated by BWA were converted to bam format using Samtools (6). Peak calling was performed with MACS2 (12) default condition and normalization on the respective Input dataset of each cell lines. Bigwig files used for visualization were generated from the fragment pileup bedGraph using the BedGraphToBigwig function.

<u>ChIP-Seq Differential Binding Analysis</u>

Differentially binding region were quantified using DiffBind 3.0 (13). Bam and narrowPeak files for each samples and bam files of the corresponding input were used. Default normalization and analysis was performed for H3K4me3 and H3K27ac. CTCF normalization and analysis was performed with the following parameters: normalize = DBA_NORM_DEFAULT, library = DBA_LIBSIZE_PEAKREADS, background = F, bREtrieve = F. Threshold of significance were set at FDR <= 0.01 and abs(LogFC) >= 1 in all conditions. Consensus differential peaksets between replicates and conditions were used for further downstream analysis and converted to Grange format using GenomicRanges R packages (14). The number of sites in each peak set was used for quantification and the generation of pie chart.

<u>ChIP-Seq Genomic Distribution and Sites Annotation</u>

Genomic distribution and annotation were performed using clusterProfiler package (15) and ChIPSeeker package (16) on the differential binding sites identified by DiffBind 3.0, using the TxDb.Hsapiens.UCSC.hg19.knownGene as reference for gene location. TSS regions were defined with a +/- 3000bp overlap during peak annotation. The regions are annotated as : 5'UTR, Promoter (<=1kb), Promoter (1-2kb), Promoter (2-3kb) are referred to as "Promoter (+/- 3kb)"; 1st Exon and Other Exon are referred to as "Exons"; 1st Intron and Other Intron are referred to as "Introns"; 3'UTR and Downstream are referred to as "Downstream" and Distal Intergenic is referred to as is.

<u>ChIP-Seq Differential KEGG Pathway Analysis</u>

Pathway analysis was performed using the annotation files from above and using the compareCluster function from the previously mentioned clusterProfiler package and the following parameters: geneCluster = genes, fun = "enrichKEGG", pvalueCutoff = 0.05, pAdjustMethod = "BH". -log(p.value) of enrichment significance was used for bar chart representation, where pathways were ranked according to geneRatio. The differential analysis was then performed by repeating these steps on 100 randomized subsets of constant CTCF sites equinumerous to the number of lost sites and on 100 randomized subsets of constant H3K27ac within TADs equinumerous to the number of gained H3K27ac within TADs. Then, the average p-value of the surveyed pathway within the 100 subsets was calculated and compared to the p-value in the lost CTCF sites or gained H3K27ac sites. P-values in subsets in which the surveyed pathways were

not detected were overestimated at 0.25, as the most significant value for which pathways are not called by the program.

ChIP-Seq and RNA-Seq Dot Plots and Correlation

Dot plots were made by combining the RNA-Seq Log2FC between CTL and CTCF+/- #1 and #2 and the logFC from DiffBind of any called peak annotated on that gene (+/- 3kb) by clusterProfiler. Spearman correlation on the dot plot were performed using the ChIP-Seq logFC and RNA-Seq log2FC of every peak colocalization with a gene. Genes associated with a peak with a DiffBind or DESEQ2 adjusted p-value > 0.05 or DiffBind LogFC or DESEQ2 Log2FC < 1 are represented in grey, while those with a LogFC/ Log2FC >= 1 or <= -1 are represented in orange and purple, respectively.

ChIP-Seq Heatmaps, Profile Plot, Tracks

Heatmaps, profile plot and tracks were generate using deepTools and samtools (6, 17). Heatmaps and Profile plot were generated using 3kb regions centered around the differential peakset identified by DiffBind and bigwig from MACS2. Both the computeMatrix and plotHeatmaps were runned with default parameter; yMax, zMax and colors were adjusted in each condition to better represent the results. Tracks were generated as profile plot of the single genomic regions of interest with a gene annotation track from IGV(18) under each figure to represent the relative location of the gene of interest.

ChIP-Seq Colocalization Analysis

Analysis of colocalization, +/- 3kb, was performed using a genomic overlap algorithm between the position of differentially binding peak sets identified by DiffBind. Observed/Expected ratios shown for colocalization of ChIP-Seq peaks were calculated using the Chi-Square formula in Microsoft Excel.

ChIP-qPCR Protocol:

The Chromatin Immunoprecipitation was done following the ChIP-Seq protocol, however using only 1mg/ml of chromatin and 30ul of beads with the antibodies listed in Supplementary Table 1. Final ChIP-product is diluted in 60ul of DNAse-free water. qPCR was performed with the ChIP product following the manufacturer protocol (GoTaq qPCR MasterMix 2X, Promega, #A600A). 2-ΔΔCT formula was used for quantification, normalized on a 2% chromatin input of each sample and compared between sites and conditions. Primers used for ChIP DNA amplification are documented in Supplementary Table 3.

Methyl Array Protocol and Data Analysis:

Methyl Array and RNA-Seq Dot Plot

Dot plot of methylation profile and RNA-Seq was generated using the Methylation LogFC between MCF10A CTCF+/- #2 and MCF10A CTL for all CpG colocalizing with a gene (+/- 2kb) with a DESEQ2 called basemean > 100. Spearman correlation was calculated using all points under these criteria.

Hi-C Data Processing and Analysis:

Quality control of reads and sequencing was assessed by FastQC (Babraham Bioinformatics). Raw sequencing read were mapped, filtered, and binned using the runHiC pipeline (19). Contact matrix were binned at 5kb and 10kb resolution and stored in ".cool" format.

Hi-C Data Processing for Pearson Correlation Analysis

Raw sequencing read were analyzed using cLoops2 pipeline (20) pre-processing program tracPre2.py, "cLoops2 pre" and "cLoops2 combine" functions, using default parameters. Pearson Correlation Coefficient was calculated using the pulled biological replicates of the pre-processing results using "cLoops2 estSim" function, using default parameters and bin size set to 5000, 10000, 500000 and 1000000.

Hierarchical TAD Calling

Hierarchical TAD calling was performed using the hiTAD function of the TADLib package (19), using the 10kb resolution contact matrix and default settings.

Domain Boundaries Colocalization Analysis

Colocalization of TAD boundaries and ChIP-Seq peak was determine as described earlier, using a simple genomic overlap algorithm between the called TAD boundaries and the differentially bound peak list generated with DiffBind, with an accepted overlap of +/- 10kb (+/- 1 contact matrix bin). Observed/Expected calculation were performed as described earlier. The same algorithm and overlap were used across samples to determine altered boundaries. TAD and subTAD boundaries present only in the CTL, but in none of both the CTCF+/- clones were defined and quantified as lost boundaries. TAD and subTAD boundaries present in both CTCF+/- clones, but absent in the CTL were defined and quantified as gained boundaries. Boundaries found in the CTL and any of the CTCF+/- clone were defined as constant boundaries.

Constant or gained boundaries were defined as adjacent to lost boundaries is the next or second to next boundaries, in any direction, is lost. Significant enrichment of the gained boundaries next to lost boundaries, compared to constant boundaries, was performed using the Chi-Square formula in Microsoft Excel.

RNA-Seq Changes at Domain Boundaries

Constant and altered boundaries' genomic location (10kb each) were annotated as previously described in the ChIP-Seq Analysis section. The average RNA-Seq log2FC of the CTCF+/- #2 against the CTL of genes colocalizing with each type of boundaries was calculated and represented by a bar chart. Statistical test of the difference between the average RNA Log2FC at each boundary was performed using a Student's T Test. Pathway analysis of altered boundaries was performed as in the ChIP-Seq section.

Hi-C 2D Heatmaps

Genome wide and Chromosome 20 heatmaps were generated using Juicer (21) representation of observed interacting reads value. 5kb resolution heatmaps were generated from h5 converted cool files using the HiCExplorer packages (22) default settings, using the hicPlotMatrix function for interaction matrix and hicCompareMatrices for comparative interaction matrix and hicConvertFormat for the cool to h5 conversion. High resolution sub5kb HiC imaging was performed with the HIFI pipeline (23), using the Markovian Recombination Method on defined subsection of the genome (around SNAI1, chr20:48,550,000-48,750,000) and default setting for this method.

Hi-C Pile-Up Plots

Pile-Up plots were generated using the cooltools package (Open Chromosome Collective), centered at the differential peak list from DiffBind (for CTCF and histone marks) or from the TADlib TAD caller (for TAD/subTAD boundaries), normalized on random background interaction and using default settings. Local interactions were map at +/- 200kb around the defined regions. Average interactions were mapped at +/-50kb around the defined region.

Insulation Score and Profile Plots

Insulation Score was calculated at 30kb resolution and outputted as a bigwig file using the FAN-C insulation command default settings (24) and the respective 10kb .cool matrix from MCF10A CTL and MCF10A CTCF+/- #2. The bigwig file was used to generate the profile plot using deepTools, previously explained in the ChIP-Seq section, at +/- 200kb around lost sites of CTCF or gained sites of H3K27ac colocalizing with TAD boundaries (+/- 10kb) or within TADs.

Supplementary Table 1: Antibodies

Antibodies	Source	Catalog Number
Rabbit Monoclonal anti-4E-BP1	Cell Signaling	#9644
Rabbit Monoclonal anti-CDH1	Cell Signaling	#3195
Mouse Monoclonal anti-CTCF	BD	#612149
Mouse Monoclonal anti-GapDH	Origene	#TA802519
Rabbit Monoclonal anti-HA-Tag	Cell Signaling	#3724
Rabbit Polyclonal anti-phospho-4E-BP1 (Ser65)	Cell Signaling	#9451
Rabbit Polyclonal anti-PARP1	Cell Signaling	#9542
Mouse Monoclonal anti-phospho-p70S6K1 (Thr389)	Cell Signaling	#9206
Rabbit Monoclonal anti-p70S6K	Cell Signaling	#2708
Mouse Monoclonal anti-Snail	Cell Signaling	#3895
Mouse Monoclonal anti-α-Tubulin	Cell Signaling	#3873
Mouse Monoclonal anti-β-Actin	SigmaAldrich	#A5316
Rabbit Polyclonal anti-CTCF	EMD Millipore	#07-729
Rabbit Polyclonal anti-acetyl-Histone H3 (Lys27)	EMD Millipore	#07-360
Rabbit Polyclonal anti-trimethyl-Histone H3 (Lys4)	EMD Millipore	#07-473
Rabbit Polyclonal anti-trimethyl-Histone H3 (Lys27)	EMD Millipore	#07-449
Rabbit Polyclonal anti-trimethyl Histone H3 (Lys9)	Diagenode	#C15410056
Goat Polyclonal anti-Rabbit-IgG	SeraCare	#5220-0458

Goat Polyclonal anti-Mouse-IgG	SeraCare	#5450-0011
Rabbit Polyclonal anti-phospho-S6 Ribosomal Protein (Ser240/244)	Cell Signaling	#2215
Goat Polyclonal anti-Rabbit-IgG with Alexa 488 fluorophore	Invitrogen	#A32731

Supplementary Table 2: RT-qPCR Primers

Name	Sequence
GapDH F	CAGCCTCAAGATCATCAGCA
GapDH R	TGTGGTCATGAGTCCTTCCA
RPL4 F	GCTCTGGCCAGGGTGCTTTTG
RPL4 R	ATGGCGTATCGTTTTTGGGTTGT
RPLPO F	TTAAACCCTGCGTGGCAATCC
RPLPO R	CCACATTCCCCCGGATATGA
SOX9 F	AGCAAGACGCTGGGCAAG
SOX9 R	GTAATCCGGGTGGTCCTTCT
JAM3 F	CCAGGATCGAGTGGAAGAAA
JAM3 R	CAGGGATGTCTTCCCCAGT
ERBB3 F	AAAGGACCAGAGCTTCAAGA
ERBB3 R	CCAGCATCATGAAAATCACT
FGFR1 F	CCTCTTCAGAGGAGAAAGAAACA
FGFR1 R	TCTTTTCTGGGGATGTCCAA
LAMA1 F	GCAAAGGCAGAACAAAGGTC
LAMA1 R	GGCCGTCGACAGTTATGAAG
SNAI1 F	ACCTGTTTCCCGGGCAATTT
SNAI1 R	CTGGGAGACACATCGGTCAG

Supplementary Table 3: ChIP-qPCR Primer

Name	Sequence
JAM +0.5kb F	CAGTGCTGTGCTCTCCAGAA
JAM +0.5kb R	AGGGCTGTGACCAAGCAG
JAM -0.5kb F	GAAGGCGATAATGCTTCCAA
JAM -0.5kb R	CAGGTCGGAGAAGGAACACT
LAMA1 +0.25kb F	AAAGCCTAAGCCTGCAAAGA
LAMA1 +0.25kb R	ATCCTGATCCACCTCGGAGT
LAMA1 +0.5kb F	CTTTAACCTCCTCGGGCTTT
LAMA1 +0.5kb R	CAGCACTGCTCGCGTAGAT
LAMA1 +1kb F	TTTGTGACTGCCTAGCCAAC
LAMA1 +1kb R	TTTTGGGGGACAACCCTAGT
SNAI1 -0.5kb F	CGTAGACTGTCTGGGCCAAT
SNAI1 -0.5kb R	AGGCTTCCATCCTCCAACTT
SNAI1 TSS F	CCCTCCATTCTCATCAGCTC
SNAI1 TSS R	CCGATAAACTCCCTTGGACA
SNAI1 +0.5kb F	GCACACCTGACATGCTGACT
SNAI1 +0.5kb R	CCCTGACCATCACAGGCTAT
SNAI1 -652 F	CGGGAGAGGCTCTGAGTGTT
SNAI1 -652 R	CTAGCCAAGAGCACCCGTTC
SNAI1 +1kb F	GATGAGGACAGTGGGAAAGG
SNAI1 +1kb R	GCCTCCAAGGAAGAGACTGA
chr19:49,345kb F	AGTGGTCCTCACCCTCACAC
chr19:49,345kb R	GATGGCAGTAGCACACAGGA

Supplementary References:

1. X. Liu *et al.*, Conditional reprogramming and long-term expansion of normal and tumor cells from human biospecimens. *Nat Protoc* **12**, 439-451 (2017).
2. I. Sirois *et al.*, A Unique Morphological Phenotype in Chemoresistant Triple-Negative Breast Cancer Reveals Metabolic Reprogramming and PLIN4 Expression as a Molecular Vulnerability. *Mol Cancer Res* **17**, 2492-2507 (2019).
3. K. Labun *et al.*, CHOPCHOP v3: expanding the CRISPR web toolbox beyond genome editing. *Nucleic Acids Res* **47**, W171-W174 (2019).
4. A. M. Bolger, M. Lohse, B. Usadel, Trimmomatic: a flexible trimmer for Illumina sequence data. *Bioinformatics* **30**, 2114-2120 (2014).
5. A. Dobin *et al.*, STAR: ultrafast universal RNA-seq aligner. *Bioinformatics* **29**, 15-21 (2013).
6. H. Li *et al.*, The Sequence Alignment/Map format and SAMtools. *Bioinformatics* **25**, 2078-2079 (2009).
7. Y. Liao, G. K. Smyth, W. Shi, featureCounts: an efficient general purpose program for assigning sequence reads to genomic features. *Bioinformatics* **30**, 923-930 (2014).
8. M. I. Love, W. Huber, S. Anders, Moderated estimation of fold change and dispersion for RNA-seq data with DESeq2. *Genome Biol* **15**, 550 (2014).
9. A. Subramanian *et al.*, Gene set enrichment analysis: a knowledge-based approach for interpreting genome-wide expression profiles. *Proc Natl Acad Sci U S A* **102**, 15545-15550 (2005).
10. H. Mi, P. Thomas, PANTHER pathway: an ontology-based pathway database coupled with data analysis tools. *Methods Mol Biol* **563**, 123-140 (2009).
11. M. Gillespie *et al.*, The reactome pathway knowledgebase 2022. *Nucleic Acids Res* **50**, D687-D692 (2022).
12. Y. Zhang *et al.*, Model-based analysis of ChIP-Seq (MACS). *Genome Biol* **9**, R137 (2008).
13. C. S. Ross-Innes *et al.*, Differential oestrogen receptor binding is associated with clinical outcome in breast cancer. *Nature* **481**, 389-393 (2012).
14. M. Lawrence *et al.*, Software for computing and annotating genomic ranges. *PLoS Comput Biol* **9**, e1003118 (2013).
15. G. Yu, L. G. Wang, Y. Han, Q. Y. He, clusterProfiler: an R package for comparing biological themes among gene clusters. *OMICS* **16**, 284-287 (2012).
16. G. Yu, L. G. Wang, Q. Y. He, ChIPseeker: an R/Bioconductor package for ChIP peak annotation, comparison and visualization. *Bioinformatics* **31**, 2382-2383 (2015).
17. F. Ramirez, F. Dundar, S. Diehl, B. A. Gruning, T. Manke, deepTools: a flexible platform for exploring deep-sequencing data. *Nucleic Acids Res* **42**, W187-191 (2014).
18. J. T. Robinson *et al.*, Integrative genomics viewer. *Nat Biotechnol* **29**, 24-26 (2011).

19. X. T. Wang, W. Cui, C. Peng, HiTAD: detecting the structural and functional hierarchies of topologically associating domains from chromatin interactions. *Nucleic Acids Res* **45**, e163 (2017).
20. Y. Cao, S. Liu, G. Ren, Q. Tang, K. Zhao, cLoops2: a full-stack comprehensive analytical tool for chromatin interactions. *Nucleic Acids Res* **50**, 57-71 (2022).
21. N. C. Durand *et al.*, Juicer Provides a One-Click System for Analyzing Loop-Resolution Hi-C Experiments. *Cell Syst* **3**, 95-98 (2016).
22. F. Ramirez *et al.*, High-resolution TADs reveal DNA sequences underlying genome organization in flies. *Nat Commun* **9**, 189 (2018).
23. C. J. Cameron, J. Dostie, M. Blanchette, HIFI: estimating DNA-DNA interaction frequency from Hi-C data at restriction-fragment resolution. *Genome Biol* **21**, 11 (2020).
24. K. Kruse, C. B. Hug, J. M. Vaquerizas, FAN-C: a feature-rich framework for the analysis and visualisation of chromosome conformation capture data. *Genome Biol* **21**, 303 (2020).
